# Optimization of antibody binding by adjusting thermodynamic stability during the maturation of antibody affinities

**DOI:** 10.1002/pro.70739

**Published:** 2026-08-06

**Authors:** Yoko Akazawa‐Ogawa, Tomonari Matsuda, Norihiko Kiyose, Nobuo Miyazaki, Tetsuo Fukuta, Motoyasu Adachi, Yuji Ito, Yoshihisa Hagihara

**Affiliations:** ^1^ Molecular Biosystems Research Institute National Institute of Advanced Industrial Science and Technology (AIST) Ikeda Osaka Japan; ^2^ Department of Environmental Engineering, Graduate School of Engineering Kyoto University Kyoto Japan; ^3^ Division of Antibody Operations ARK Resource Co., Ltd. Kumamoto Japan; ^4^ JSR Bioscience and Informatics R&D Center (JSR BiRD) JSR Corporation Kawasaki‐shi Kanagawa Japan; ^5^ Institute for Quantum Life Science National Institutes for Quantum Science and Technology (QST) Chiba Japan; ^6^ Graduate School of Science and Engineering Kagoshima University Kagoshima Japan

**Keywords:** antibody maturation, antibody repertoire analysis, single‐domain antibody, somatic hypermutation, thermodynamic stability

## Abstract

Somatic mutations and antibody clone selection occur in B‐cell hyper‐evolution over extremely brief periods of time. Herein, we developed a technique for antibody screening by immunizing alpacas with antigens and observing antibody sequence transitions over time, a method we named Tracking the Evolution of Antibodies over time. This technique allows the observation of sequence transitions of somatic mutations in antibody populations originating from the same genome. The accumulation of somatic mutations was significantly associated with enhanced antigen‐binding capacity and reduced stability in clusters. In comparison with the first clone to emerge in this cluster, numerous clones exhibited a decline in thermal stability, with a maximum variation of 21°C. Somatic mutations within the clusters demonstrating high similarity were observed to be concentrated in CDR‐1, CDR‐2, and FR‐3. This suggests that these mutations have a significant impact on binding capacity and stability. However, the correlation between antigen binding capacity and stability was insignificant and weak. The thermal stability of antibodies correlated with acid and alkali resistance, with clones exhibiting lower thermal stability demonstrating higher acid and alkali resistance in antigen–antibody complexes. The results indicate that in the antibody selection process, the strength of antigen binding involves optimizing stability, with antibodies with more flexible structures being selected.

## 1 INTRODUCTION

The industrial utility of antibodies relies on high affinity, specificity, and stability. However, modifying a protein's sequence to enhance its function often leads to protein instability and conformational changes (Beadle & Shoichet, 2002; Bloom et al., 2005). This is particularly evident in studies on the direction of functional evolution of proteins (Thomas et al., 2010; Wang et al., 2002). The trade‐off between activity and stability, commonly observed in enzymes, has been reported in antibodies (McConnell et al., 2014; Mishra & Mariuzza, 2018; Nishiguchi et al., 2022; Tokuriki & Tawfik, 2009). However, the complexity of this phenomenon in antibody evolution is not yet fully understood due to the limited number of clones experimentally obtained through successive somatic hypermutation in vivo. The variable regions of antibodies undergo high‐frequency mutagenesis, termed somatic hypermutation, in which mutations accumulate mainly in the complementarity‐determining regions (CDRs) (Leighton et al., 2018; Yu et al., 2023). Typically, mutations in hypervariable CDRs negatively impact antibody stability (Julian et al., 2017). In our recent work, we successfully visualized changes in antibody sequences during the immunostimulatory process. This approach has enabled the observation of somatic hypermutation in antibody populations with the same genomic ancestry over time (Matsuda et al., 2023).

Generally, the frequency of somatic genetic variation in the light chain's variable region (VL) domain is less frequent than in the heavy chain's variable region (VH) domain of heavy chains (Kosmas et al., 1998). However, accurate assessments of binding capacity and stability can only be made when light and heavy chains are combined correctly. The use of the VH domain of camelid heavy‐chain antibodies (VHH) eliminates this issue, making them particularly suitable for studies on protein structure–function relationships related to somatic hypermutation. In this study, sequence variation in VHH antibodies during antigen immunization processes was investigated using a method we named Tracking the Evolution of Antibodies over time (TEA‐time), with particular emphasis on their effects on functionality and stability.

## 2 RESULTS

### 2.1 Somatic hypermutation in antibody cluster

**FIGURE 1:**
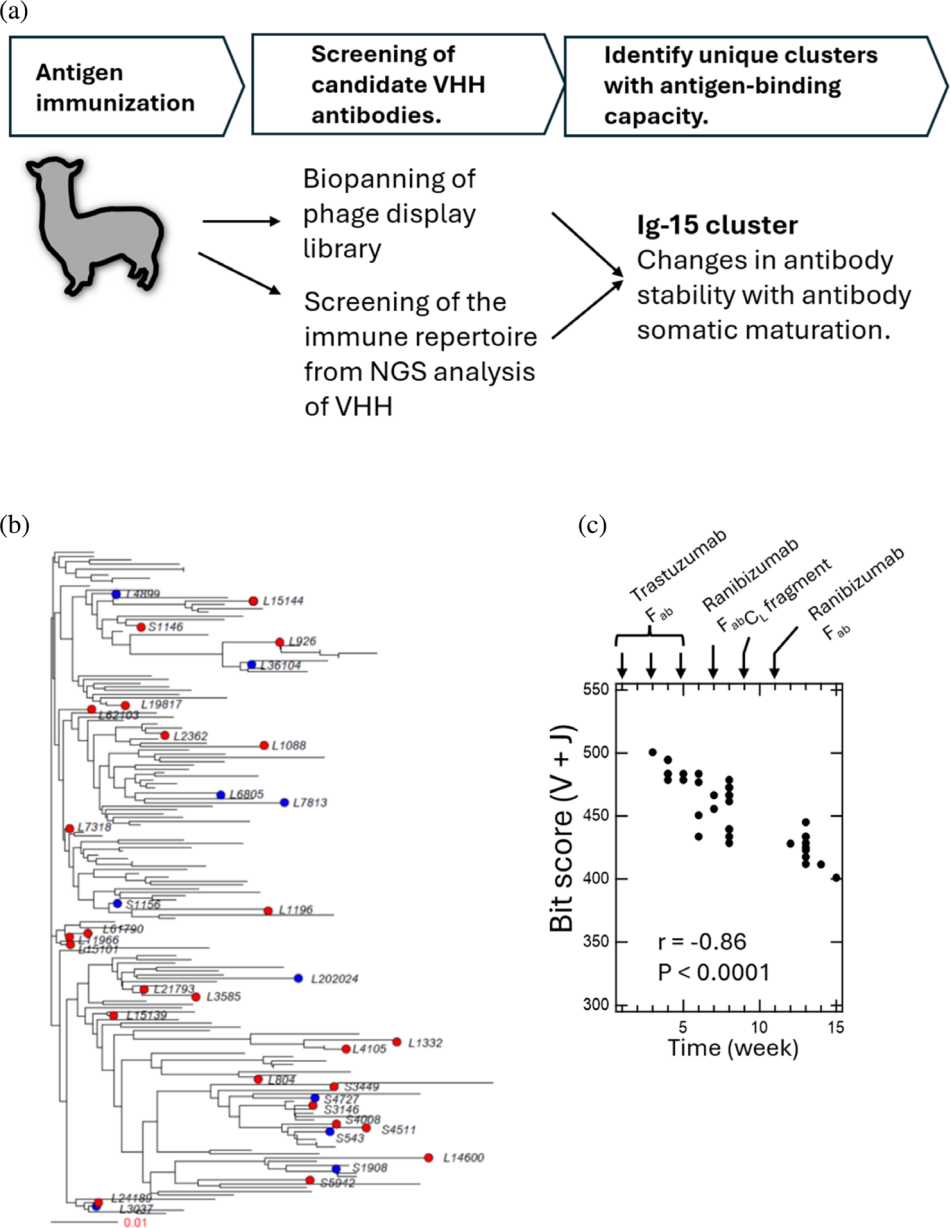
Analysis of the resulting antibody repertoire. (a) Subsequent to the administration of antigens, NGS libraries of VH domain of camelid heavy‐chain antibodies (VHH) were constructed from 15 blood samples collected weekly. Over the course of the screening process for antigen‐binding antibodies, the presence of the Ig‐15 cluster, which originates from IGHV3S53‐IGHJ4, was confirmed. Multiple antibodies derived from the Ig‐15 cluster were identified among those obtained via phage display screening. (b) Phylogenetic tree of Ig‐15 cluster. The antibodies evaluated for stability in this study are indicated by circles. Blue clones were obtained via phage display screening, while red clones were obtained only via NGS analysis (Tracking the Evolution of Antibodies over time). (c) Bit scores for each antibody within Ig‐15 cluster are plotted. The antigen immunization schedule is displayed at the top of the graph.

The cluster Ig‐15 of F_ab_‐binding antibodies, previously identified in our study, was derived from the IGHV3S53‐IGHJ4 genome, which consists of a 354‐base gene sequence showing more than 88% identity (Figure 1a) (Matsuda et al., 2023). The Ig‐15 cluster contained 178 VHH antibodies. The 35 antibodies used within the clusters in this study were assigned cluster numbers in a phylogenetic tree (Figure 1b). The selection of clones within the Ig‐15 cluster was performed based on cumulative relative frequency, inclusion of phage display‐derived clones, and diversity across phylogenetic subclusters and weeks of first appearance (Table S1). The phylogenetic tree was divided into three subclusters according to its branching structure (Figure S1). Representative clones were selected from clones ranked by cumulative relative frequency to prioritize highly frequent sequences while ensuring coverage of the major subclusters and weeks of first appearance. In addition, 10 phage display‐derived clones were included. Clones with identical amino acid sequences were represented by a single clone for subsequent analyses.

**FIGURE 2:**
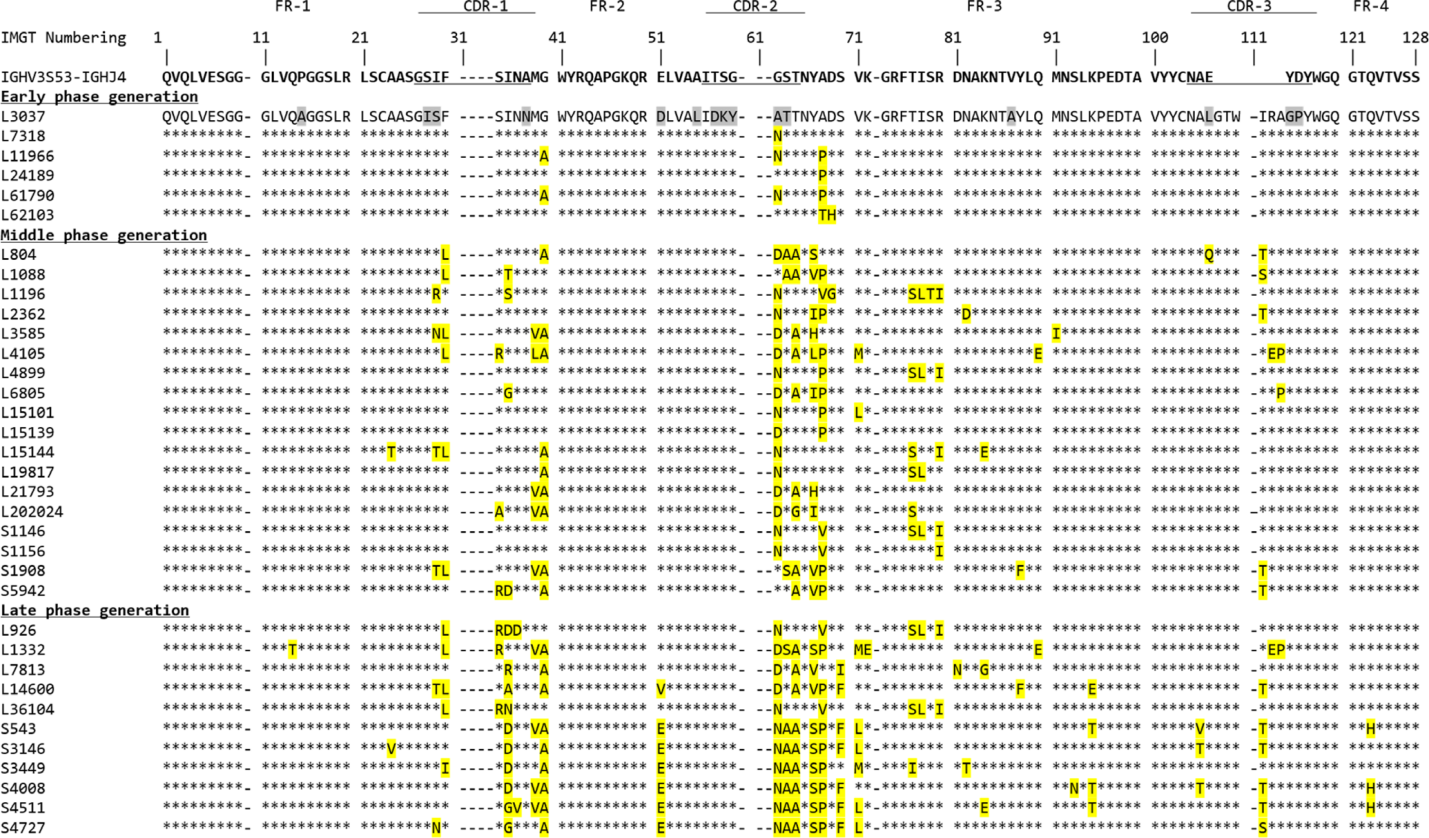
Somatic mutations in the maturation sequence of the cluster derived from IGHV3S53‐IGHJ4. Thirty five 35 VH domain of camelid heavy‐chain antibodies, derived from the Ig‐15 cluster, were selected for evaluation of their affinity and stability. The complementarity‐determining region (CDR) regions and their numbering follow the international ImMunoGeneTics information system (IMGT) scheme. L3037 was the first antibody to appear in this cluster following the start of antigenic immunity. The gray highlighting of amino acids in L3037 denotes positions that have undergone mutation relative to IGHV3S53–IGHJ4. The yellow highlighting of amino acids denotes the positions that have undergone mutation in relation to L3037.

Sequence analysis of the Ig‐15 cluster revealed highly conserved CDR3 junction features and stepwise diversification patterns, strongly supporting a predominantly monoclonal origin of this cluster (Figure S2). The bit score values (V + J) of the selected antibodies decreased over time and correlated strongly with the timing of immunization (Figure 1c). This suggests that antibody mutations accumulated as immunization progressed. The first clone, L3037, emerged at Week 3, showing mutations in 14 amino acids compared to the IGHV3S53‐IGHJ4 amino acid sequence, excluding mutations in the D residue. The 33 evolved VHH antibodies contained up to 16 amino acid mutations in their framework and CDRs compared to L3037, which accumulated sequentially during successive cycles of mutation and selection. In particular, CDR‐1 and CDR‐2 contained numerous mutations, which are assumed to be significantly involved in optimizing binding capacity (Figure 2, Table S2).

### 2.2 Crystal structure analysis of antigen–antibody complexes

Alpacas were immunized by administering F_ab_ antigen at 2‐week intervals. A VHH antibody library was constructed using blood samples collected 9 weeks after immunization, and phage display screening was performed. L202024 was one of the clones obtained via this screening process. Previously, we conducted next‐generation sequencing (NGS) and constructed a molecular phylogenetic tree of antibody sequences, which were obtained using the same immunization material. The dataset was subjected to a grouping process, wherein sequences were categorized based on their length and combinations of IGHV and IGHJ types. To facilitate the analysis, minor sequences with maximum frequency = 1 were excluded from the data of each group (Matsuda et al., 2023). The L202024 clone, an antibody obtained via phage display screening, originated from IGHV3S53‐IGHJ4 and showed high homology with the Ig‐15 cluster (Figure 2). However, as the L202024 clone was a minor sequence with a maximum frequency of 1, it was excluded prior to cluster classification and was not included in the Ig‐15 cluster to simplify the cluster analysis; however, as it was discovered early in this research, it was selected for crystallographic analysis.

**FIGURE 3:**
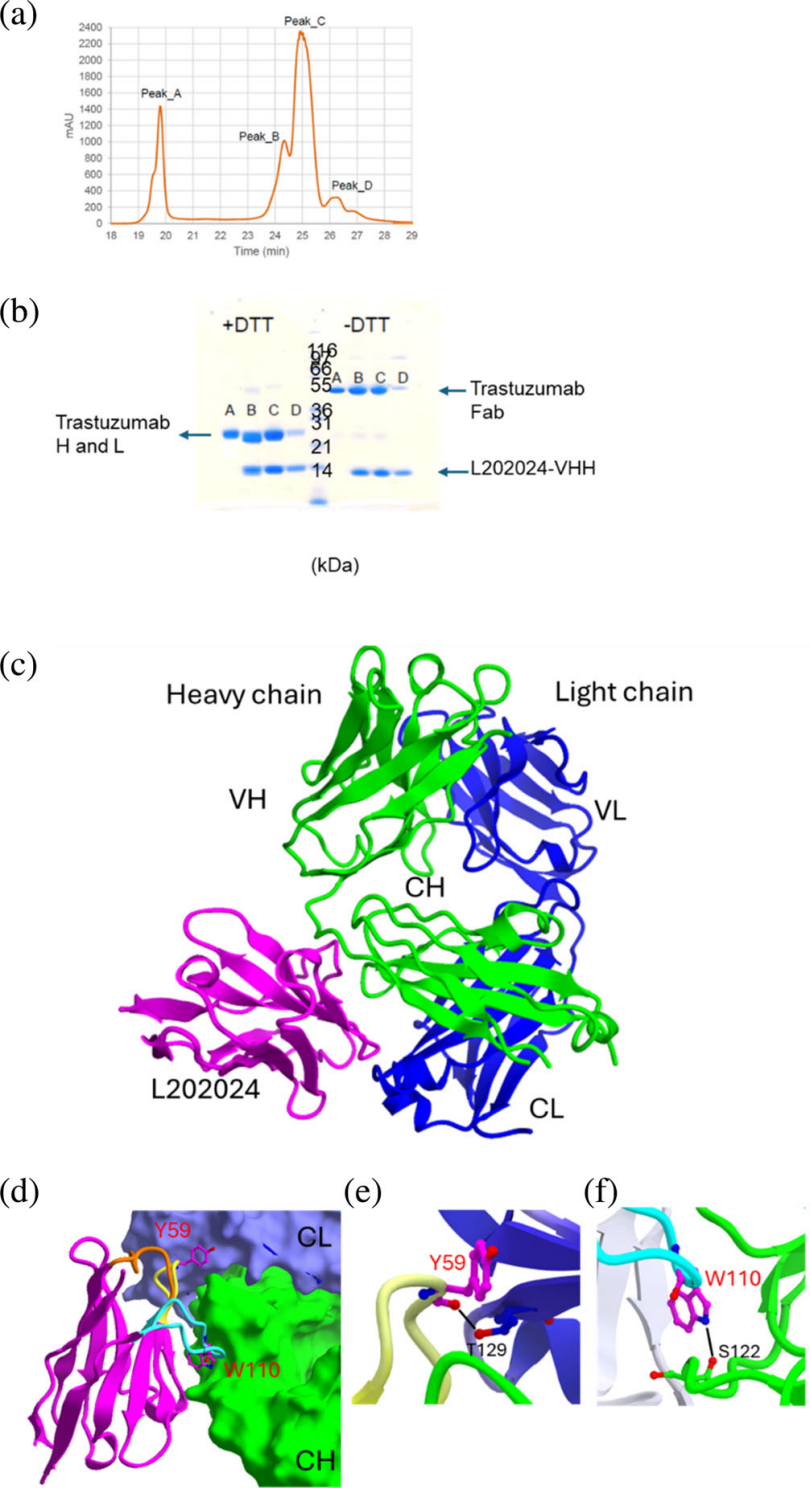
Crystal structure of the L202024 and Trastuzumab‐F_ab_ complex. (a) Elution spectrum at 280 nm of a 1:1 mixture of Trastuzumab‐F_ab_ and L202024 purified on a Resource S column. (b) The fractions eluted from a Resource S column were then subjected to SDS‐PAGE, with and without dithiothreitol (DTT). The complex fractions, C were concentrated to a final concentration of 12.5 mg/mL. (c) Ribbon structure of L202024 and Trastuzumab‐F_ab_ (PDB ID: 1N8Z). The L202024 domain is shown in magenta, and the VH‐CH1 and VL‐CL domains of Trastuzumab‐F_ab_ are shown in green and blue, respectively. (d) L202024 (ribbon structure) and the CH and CL domains of Trastuzumab‐F_ab_ (surface structure). The CDRs of the L202024 clones were shown by CDR‐1 (orange), CDR‐2 (yellow) and CDR‐3 (light blue). Y59 and W110 were shown in the ballstick model. Y59 (yellow) of CDR‐2 and the T129 of CL domain (e), as well as W110 (light blue) of CDR‐3 and the S122 (f) of CH1 domain, form hydrogen bonds. VHH, VH domain of camelid heavy‐chain antibodies.

Crystallographic analysis of the trastuzumab‐F_ab_‐L202024 complex revealed that the epitope was mainly present in the CH1 domain (Figure 3a–c and Table S3). The paratope of L202024 comprised multiple residues distributed mainly across the CDRs and parts of the framework regions that were in direct contact with the antigen. Among these, W110 in CDR‐3 and Y59 in CDR‐2 were identified as key interacting residues. W110 interacted with the CH1 domain, whereas Y59 interacted with the CL domain (Figure 3c–f). The nitrogen atom in the side chain of W110 and the oxygen atom in the main chain of Y59 formed hydrogen bonds with the trastuzumab heavy chain and light chain, respectively (Table S4). Based on the crystal structure, interacting residues were further evaluated according to changes in accessible surface area (ASA) upon complex formation. Residues exhibiting an ASA decrease of ≥1 Å^2^ were defined as interface residues contributing to the paratope. In L202024, residues Y59 and W110 showed notable decreases in ASA upon complex formation. Using this criterion, 20 residues were identified as part of the paratope, including 10 in the CDRs and 10 in FR‐2 and FR‐3 (Table S5).

On the antigen side, residues in the CH1 and CL domains that showed significant ASA reductions were defined as epitope residues. A larger reduction in ASA was observed in the CH1 domain (6.36%) compared with the CL domain (2.18%), indicating that L202024 predominantly interacted with the CH1 domain (Table S6).

### 2.3 Evolved antibody variable domains exhibited a trade‐off between affinity and stability

In order to evaluate the correlation between antigen binding and stability in Ig‐15‐derived VHHs, 35 types of VHH were produced in *Escherichia coli* (Matsuda et al., 2023). Among the tested clones, L3037, L1332, L14600, S543, S3146, S3449, S4008, S4511, and S4727 could not be completely regenerated following acid (pH 2.5) washing of the sensor chip. Therefore, a capture method was developed to assess their binding capacity. In this approach, a polyclonal anti‐human F_ab_ antibody was immobilized on a CM5 sensor chip, followed by antigen binding, and the binding capacity of each VHH at various concentrations was measured. To examine whether VHH antibodies belonging to the Ig‐15 cluster recognize the same epitope, SPR‐based epitope competition experiments were performed. Trastuzumab F_ab_ was captured on an anti‐F_ab_ polyclonal antibody–immobilized sensor chip, followed by VHH clone L1332. Subsequent injection of other VHH clones (L804, L7318, L6805, L15144, and L21793) substantially reduced binding in the presence of pre‐bound L1332. Because these clones were derived from both the same and different subcluster of the Ig‐15 phylogenetic tree, the observed competition indicates that Ig‐15 cluster antibodies predominantly recognize the same or highly overlapping epitope on the F_ab_ antigen (Figure S3).

**FIGURE 4:**
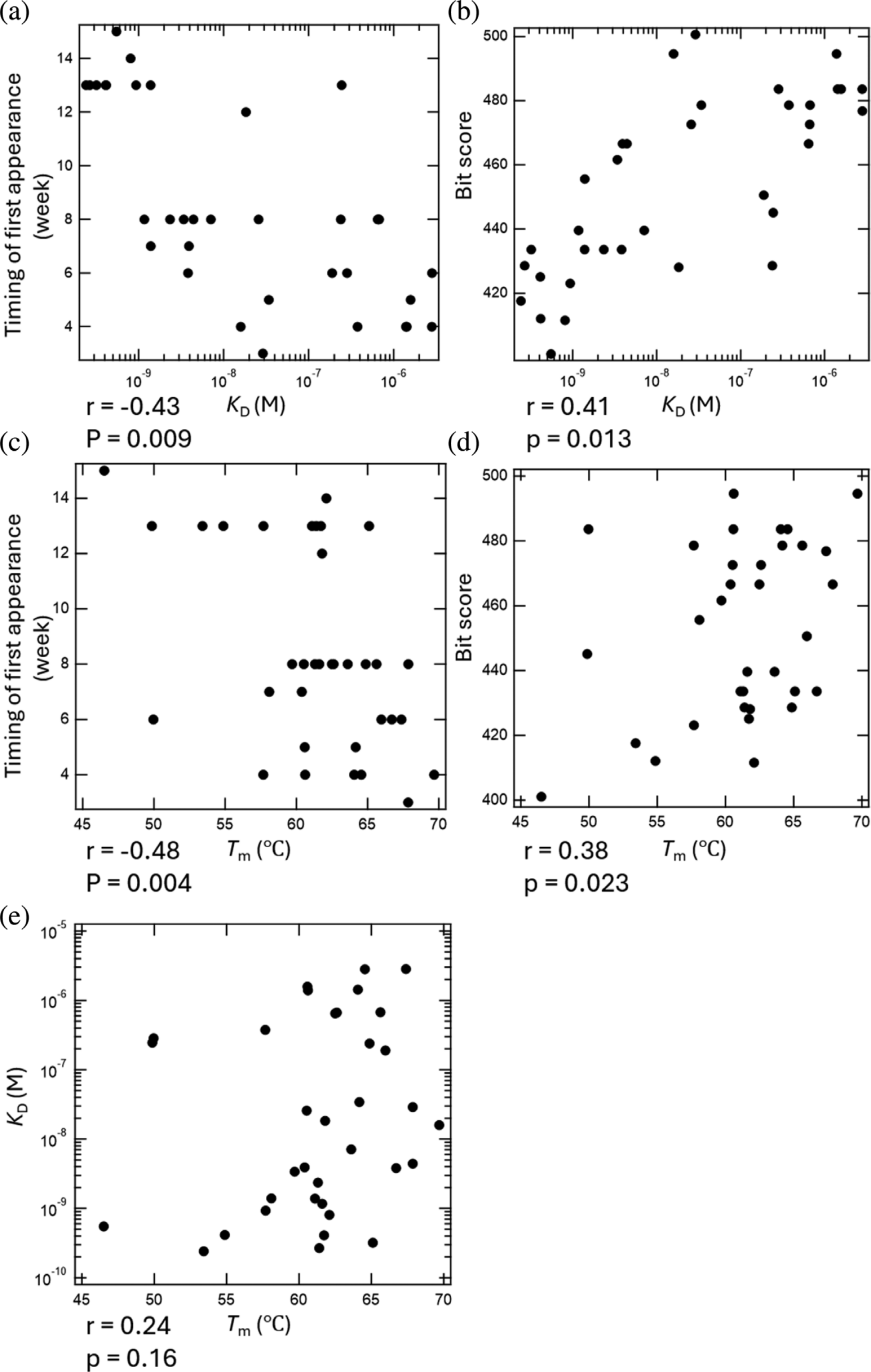
Comparison of each VH domain of camelid heavy‐chain antibodies (VHH) in terms of binding affinity and thermodynamic stability. The antigen affinity and stability of VHH variants within the single cluster shown in Table S2 were evaluated. The bit score is a numerical value quantifying similarity to the IGHV3S53‐IGHJ4 sequence, with higher values indicating greater similarity. An increasing trend in binding affinity was observed following antigen immunizations and somatic hypermutation (a and b). Thermal stability exhibited a decreasing trend (c and d), yet no significant correlation was observed between thermal stability and affinity (e).

Binding affinity was associated with the timing of blood sampling and the accumulation of mutations (Figure 4a,b). The binding capacity analysis yielded results indicating that S3449 (*K*_D_ 0.24 nM), the clone demonstrating the highest binding capacity, exhibited a 120‐fold increase in binding capacity in comparison with that of L3037 (*K*_D_ 28.9 nM), the earliest clone in the Ig‐15 cluster. Compared to that of L24198 (*K*_D_ 1.36 μM), which exhibited the lowest binding capacity in the cluster, S3449 demonstrated a 5958‐fold increase in binding capacity (Figures S4 and S5, and Table S2). Correlation analysis was performed between the bit score and kinetic parameters (*k*_a_ and *k*_d_) obtained from the 1:1 binding analysis of the 21 VHH clones. No correlation was observed between the bit score and *k*_a_. In contrast, a significant correlation was observed between the bit score and *k*_d_ when two early appearing clones were excluded from the analysis (Figure S6), suggesting that the accumulation of mutations during affinity maturation is associated with slower dissociation kinetics.

Antibody stability was analyzed based on changes in SYPRO Orange fluorescence intensity (Figure S7). Stability was correlated with both the timing of blood collection and the bit score value, with the accumulation of mutations during the immunization process generally leading to decreased antibody stability (Figure 4c,d). For example, L3037, which closely resembled the germ‐line sequence, had a *T*_m_ value of 67.9°C. In contrast, L14600, which exhibited the lowest bit score value, had a *T*_m_ value of 46.5°C, indicating a substantial decline in stability between these clones. However, no significant correlation was observed between the binding affinity and stability, suggesting that affinity maturation does not necessarily require a loss of stability (Figure 4e).

### 2.4 Low stability antibody affects acid and alkaline tolerance of antigen–antibody complexes

**FIGURE 5:**
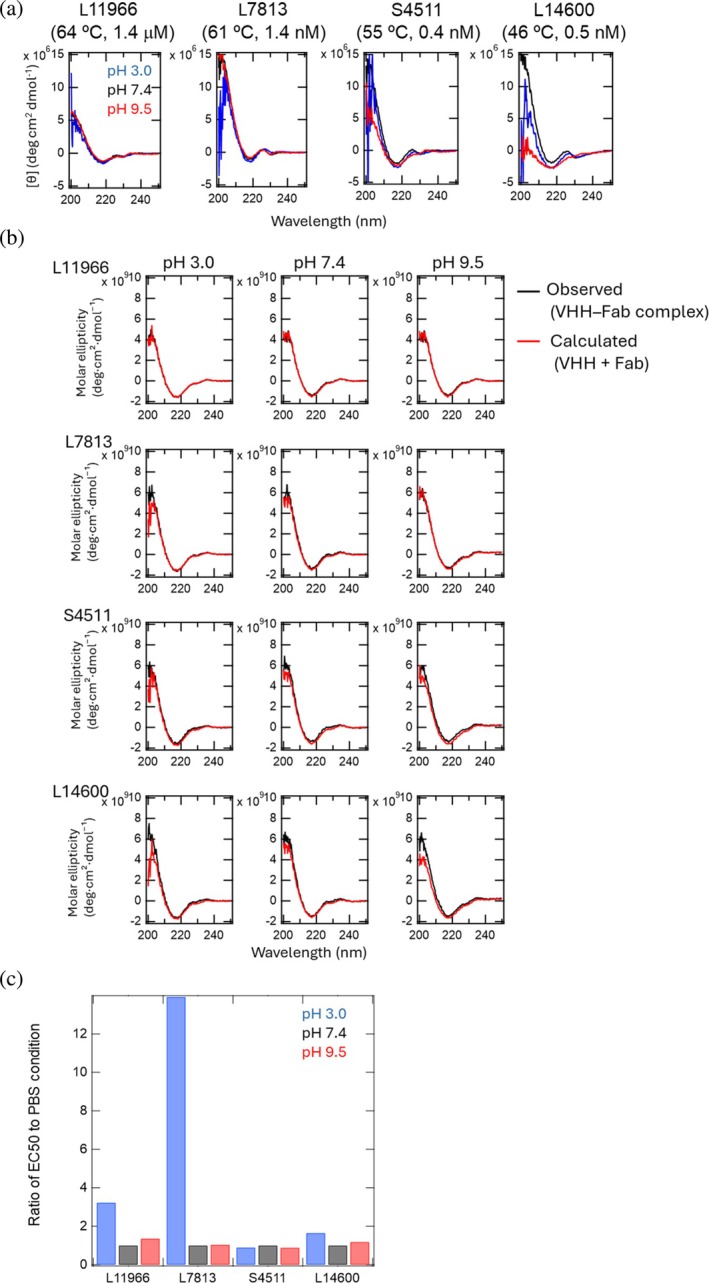
Assessment of acid and alkali resistance of antibodies and antibody–antigen complexes. (a) Circular dichroism (CD) spectra were recorded for VH domain of camelid heavy‐chain antibodies (VHH) alone (30 μM) in solutions at pH 3.0, 7.4, and 9.5. (b) pH tolerance of F_ab_ antigen and F_ab_–VHH antibody complexes. CD spectra were recorded for F_ab_ alone (6.2 μM) and F_ab_–VHH complexes (4.9 μM) in solutions at pH 3.0, 7.4, and 9.5. The calculated spectrum was generated by summing the CD spectra of VHH and F_ab_ measured separately under identical conditions. (c) ELISA of L11966, L7813, L14600, and S4511 with Trastuzumab‐F_ab_ antigen. Solvents at pH 3.0, 7.4, and 9.5 were treated following the formation of antigen–antibody complexes. Subsequently, the plates were washed, after which residual VHH antibodies were detected. The concentration of VHH antibodies exhibiting 50% activity (denoted as EC50) was utilized as an indicator of the effect of acid or alkaline treatment.

The stability of antibodies alone or in antigen–antibody complexes was assessed by evaluating their acid and alkaline resistance using CD spectroscopy and enzyme‐linked immuno‐sorbent assay (ELISA). L11966 (*T*_m_ 64.1°C) and L7813 (*T*_m_ 61.0°C) did not exhibit structural alterations at pH 3.0 and pH 9.5, as their CD spectra remained nearly identical to those observed at pH 7.4. On the other hand, L14600 (*T*_m_ 46.5°C) and S4511 (*T*_m_ 54.9°C) showed changes in the CD spectra, especially the β‐sheet‐derived spectra around 217 and 225 nm, under pH 3.0 and pH 9.5, suggesting changes in their secondary structure (Figure 5a). To examine whether pH tolerance could be explained solely by intrinsic thermal stability, additional analyses were performed on a subset of VHH antibodies. When antibody stability was assessed in the absence of antigen, clones L1332 and L4727, which exhibited *T*_m_ values above 65°C, showed high pH tolerance. By contrast, clone L3449, despite having a relatively low *T*_m_ of 53.4°C, also exhibited pH tolerance (Figure S8A). These results indicated that the pH tolerance of the isolated antibodies cannot be explained solely by their intrinsic thermal stability. In contrast, pH‐dependent CD analysis of F_ab_–VHH complexes revealed that the formation of the antigen–antibody complex improved the structural stability under acidic and alkaline conditions, even for antibodies that were destabilized when analyzed in isolation (Figure 5b).

The stability of the VHH antibody–antigen complex was evaluated for acid and alkaline resistance using ELISA. F_ab_ antigens were immobilized on plates, and VHH antibodies were added. After washing with phosohate‐buffered saline containing 0.02 % Tween (PBS‐T), the plates were treated with acid, alkali, or PBS. After 15 min at room temperature, the plates were washed again with PBS‐T, and the amount of VHH antibodies remaining was quantified. The effect of antibody dissociation by each solvent was calculated and compared based on half maximal effective concentration (EC50, the VHH concentration that binds to half the F_ab_ at equilibrium) under acidic or alkaline washing conditions (Figure S8B). The results showed that the antibodies with high thermostability (L7813 and L11966) dissociated from the antigens under acidic conditions. In contrast, the antibodies with lower thermostability (L14600 and S4511) exhibited minimal dissociation, suggesting that these antibody–antigen complexes are highly resistant to acid and alkali treatment (Figure 5c). Notably, the pH tolerance inferred from CD measurements of the isolated antibodies did not fully correlate with the functional pH tolerance for antigen binding, as assessed using ELISA. These results suggest that antibodies with low stability as single molecules may exhibit significantly enhanced stability upon forming complexes with antigens.

## 3 DISCUSSION

The maturation process of antibodies can be defined as an iterative cycle of mutation and selection, which enhances the affinity of antibodies for their target antigens (Kim et al., 1981; Tonegawa, 1983). The VHH binds to antigens in a single‐domain format and can be produced rapidly, efficiently, and cost‐effectively using an *E. coli* expression system. VHH antibodies are particularly suitable for studying the affinity maturation process, as they do not require combinatorial pairing with light chains. Furthermore, analyzing antibody sequence changes during the maturation process via antigen immunization and predicting which antibodies will react to the immunized antigen without in vitro screening provides insights into many antibodies derived from the same V‐J combination (Matsuda et al., 2023). Combining VHH antibodies with such sequence analysis enables detailed elucidation of the maturation process. Additionally, in this study, the immunization schedule involved a switch in antigens, and the emergence of VHH antibody variants ceased when CL immunization was performed at Week 8 (Figure 1c). This was likely due to the lower immunogenicity of the CL domain compared with that of the F_ab_ antibodies.

The decrease in stability observed during antibody maturation is similar to that reported for enzymes and scFv (Figure 4c,d) (Nishiguchi et al., 2022; Oda, 2022; Tokuriki & Tawfik, 2009) and reflects a common trade‐off between performance and stability (Figure 4e). The selection criteria for antibodies presented to B cells are their ability to bind antigens and maintain structural integrity on the B cell surface. Therefore, if an antibody can retain its structure at physiological temperature, stability is not thought to be a primary selection factor. This study examined the function and stability of VHH domains and found only a weak correlation with the overall decrease in antibody stability during the in vivo maturation process. However, the decrease in antibody stability may increase protein flexibility, enabling subtle structural adjustments upon antigen binding and ultimately leading to the formation of a more stable antibody–antigen complex. Previously, we investigated the effect of cysteine depletion in VHH antibodies on antigen‐binding activity. We found that the reduced stability resulting from the deletion of the disulfide bond led to an increase in binding affinity (Akazawa‐Ogawa et al., 2016). Thus, random mutations introduced into the VHH domain during the somatic mutation process may reduce antibody stability, thereby increase molecular flexibility and enhance antigen‐binding capacity. However, in the present study, no strong correlation was observed between binding affinity and stability. We believe the observed correlation between the bit score and stability may reflect a trade‐off arising from the increased mutational burden during sequence‐space exploration during affinity maturation. However, for the two clones, L14600 and S4511, a decrease in thermal stability was accompanied by an increase in the stability of the antigen–antibody complex. These cases suggest that structural flexibility resulting from reduced stability may influence binding properties; however, this remains purely speculative and requires further investigation. The lower solvent tolerance exhibited by S4511 and L14600 compared with that of L19600 and L7813 may be attributed to the increased number of E residues. Negatively charged D/E residues become uncharged in acidic conditions. Therefore, S4511 and L14600 may have undergone a change in surface charge and denaturation under acidic conditions. Antigen–antibody complexes exhibit greater stability than antibodies alone (Legler et al., 2017; Oda, 2022). Despite exhibiting low solvent resistance individually, S4511 and L14600 demonstrated high acid resistance within the complex (Figures 5c and S8B). This phenomenon may be attributable to the structural flexibility of these VHHs, suggesting that minor changes occur in the three‐dimensional structure of the VHH antibody upon antigen binding. CDRs may adopt a conformation better suited to the antigen, thereby becoming less susceptible to denaturation by the solvent. Alternatively, regions prone to destabilization by the solvent may be masked by interactions with the antigen, thereby reducing their exposure to the solvent and improving solvent resistance. CD analyses indicated that the formation of the F_ab_–VHH complex can enhance structural stability at extreme pH values, even for antibodies that are destabilized when analyzed in isolation. This discrepancy likely reflects differences in the properties evaluated by each method: CD primarily reports global structural stability, whereas ELISA assesses antigen‐binding activity. Consequently, antibodies with lower intrinsic thermal stability may retain their binding activity if antigen binding stabilizes the paratope or binding interface.

Phage display screening is widely employed to isolate antibodies from both immune and synthetic libraries and is particularly valuable for the selection of VHH antibodies. In our studies, this method has been used with immune or naïve libraries to obtain VHH antibodies capable of binding to a variety of antigens. However, antibodies obtained through biopanning, especially from immune libraries, often exhibit limited diversity, with many recovered sequences being highly similar to each other (Turner et al., 2016). The diversity of phage display libraries may be more constrained than that of natural immune repertoires, owing to factors such as improper folding of antibody fragments or unintended selection biases toward specific sequences within *E. coli* (Almagro et al., 2019). Moreover, previous studies have reported pronounced imbalances in expression frequency within immune libraries, whereby certain antibody groups are highly expressed while others are present at very low levels (Turner et al., 2016). Consequently, antibodies with high binding capacity but low expression frequency are less likely to be recovered through phage display screening.

Our ELISA studies showed that high‐affinity and low‐stability antibodies that emerged in the late immunization phase were more stable upon antigen binding and less likely to dissociate under acidic or alkaline conditions (Figure 5c). Such clones are considered to be unable to recover phages during acidic or alkaline treatment in phage display screening. Thus, antibodies that are difficult to dissociate after antigen binding are likely to result in fewer positive clones in phage display screening. Although phage display is a highly effective screening technique, it is essential that experimental protocols are carefully designed to ensure the identification of high‐affinity antibodies, especially when screening from immune libraries obtained from animals.

The L202024 clone identified by phage display was expressed only once in the immune samples and was not classified as part of the cluster Ig‐15 (Matsuda et al., 2023). However, its gene sequence showed high similarity to sequences within cluster Ig‐15. The alignment of the amino acid sequence of the VHH antibody within cluster Ig‐15 revealed a high similarity to L202024 (Figure 2).

In this study, we did not determine the crystal structure of a representative antibody formally assigned to the Ig‐15 cluster; however, its epitope was likely to be similar to that recognized by L202024, primarily within the CH1 and CL domains. The majority of antibody mutations observed in cluster Ig‐15 were concentrated in CDR‐1, CDR‐2, and FR‐3. Structural analysis of L202024 revealed that these mutation sites were located on the antibody surface. This finding suggests a particular impact on antigen interactions within the antigen‐binding groove (Figure S9). VHHs show a clear preference for the recognition of concave protein epitopes, while their recognition of protruding epitopes and small molecules is low (De Genst et al., 2006; Henry & MacKenzie, 2018). Certain “concave” regions of proteins are far more accessible to VHHs than to conventional antibodies. In this study, portions of the CDR‐1, CDR‐2, and FR‐3 variable sites of the Ig‐15 VHH were found to contact the concave surface of the antigen. This finding suggests that the evolution of antibodies may be focused on specific regions of antigens.

In vivo, a broader range of mutational events may occur beyond those identified in the present analysis. The observed accumulation of mutation sites may be attributed to the detection of viable B cells that received survival signals after antigen‐based selection. Furthermore, in the cluster analysis, CDR‐3 similarity was excluded from the clustering index, but the frequency of CDR‐3 mutations remained low. This suggests that within clusters, affinity maturation leading to enhanced antigen binding capacity is affected to a greater extent by mutations in CDR‐1 and CDR‐2 rather than CDR‐3. Mutations in the maturation process do not occur randomly within the V gene but are concentrated at specific sites in CDR‐1 and FR‐3 (Tomlinson et al., 1996). The mutational insertion sites observed in this study (particularly CDR‐1, CDR‐2, and FR‐3) were considered to be mutational hotspots.

In the present study, the effect of somatic hypermutation on the stability of VHH antibodies was investigated. Our findings suggest that mutations introduced into germline sequences contribute to increased antigen‐binding affinity but often lead to reduced stability. Nevertheless, high‐affinity antibodies are not inherently unstable. Furthermore, these results indicate that increased conformational flexibility may play a role in enhancing antigen‐binding affinity.

## 4 MATERIALS AND METHODS

### 4.1 Alpaca immunization and VHH screening

An alpaca was immunized with 1.0–2.8 mg human IgG fragments, including F_ab_ from trastuzumab (Genentech, San Francisco, CA, USA), ranibizumab (Genentech), and human κ CL, every 2 weeks for a total of six treatments (Figure 1). Complete and incomplete Freund adjuvants (Becton, Dickinson and Company, Franklin Lakes, NJ, USA) were used after the first and subsequent immunizations, respectively. Blood collection (30–50 mL) began just before the first antigen injection and was performed weekly for 14 weeks for IgG experiments. Lymphocytes were purified from 30 to 50 mL of blood by the Ficoll–Plaque method using Leucosep tubes (Greiner Bio‐One, Frickenhausen, Germany). Purified lymphocytes were homogenized in RNAiso Plus (Takara Bio Inc., Kusatsu, Japan) and stored at −80°C until use. Total RNA was extracted from alpaca lymphocyte homogenate in RNAiso Plus according to the manufacturer's protocol. Phage display libraries were prepared from the ninth blood sample and candidate antibodies were obtained by biopanning using Trastuzumab‐F_ab_. The method for constructing the alpaca VHH phage library and biopanning against IgG fragments was the same as described previously. NGS libraries were generated from all blood samples and screened using our antibody sequence evolutionary tracking method, yielding the Ig‐15 cluster of IGHV3S53‐IGHJ4 and within 40 bases of the genome sequence at 354 bp in length. The VHH antibodies belonging to the Ig‐15 cluster analyzed in this study were obtained from previously published NGS datasets reported (Matsuda et al., 2023). The methods of NGS library preparation and NGS analysis were the same as described previously. The clone nomenclature reflects the hinge length of the parental heavy‐chain antibodies: “L” denotes clones derived from heavy‐chain antibodies with a long hinge, whereas “S” denotes clones derived from antibodies with a short hinge.

The phylogenetic tree of VHH clones in the Ig‐15 cluster was constructed based on sequence similarity and further divided into three subclusters according to its branching structure (Figure S1). Clone selection was performed based on (i) cumulative relative frequency, (ii) inclusion of phage display‐derived clones, and (iii) diversity across different phylogenetic subclusters and weeks of first appearance. Clones were first ranked in descending order of cumulative relative frequency. Representative clones were then selected to prioritize highly frequent sequences while ensuring coverage of the major phylogenetic subclusters and weeks of first appearance. In addition, phage display‐derived clones were included in the selection. Clones with identical amino acid sequences were not analyzed separately; instead, a single representative clone was used for subsequent analyses. Clones selected but excluded from further analysis due to experimental limitations were designated as “selected but excluded.” All clones in the Ig‐15 cluster, together with their relative frequencies, subcluster assignments, selection status, and selection rationale, are listed in Table S1.

### 4.2 VHH expression and purification

All clones of VHH were generated via gene synthesis (Eurofins Genomics) using codon frequencies optimized for *E. coli* and were added into the pAED4 plasmid. VHH proteins were expressed by transfecting VHH‐encoding plasmids into *BL21 (DE3) pLy*S, and they accumulated in inclusion bodies. The latter was dissolved in a mixture of 4 M guanidine HCl, 10 mM dithiothreitol (DTT), and 10 mM Tris–HCl (pH 8.5). The solution was left to stand at 25°C for 3 h. A HisTrap HP column (Cytiva) equilibrated with 8 M urea, 10 mM Tris–HCl (pH 8.5), and 0.5 M NaCl was used to purify the crude VHH proteins, and the latter was eluted with imidazole. The samples were subjected to air oxidation at 4°C overnight and dialyzed against 10 mM Tris–HCl (pH 8.0). Then, 1/10 volume 1 M sodium acetate (pH 4.7) was added to the samples, which were dialyzed against 10 mM sodium acetate (pH 4.7). A Resource S cation‐exchange column (Cytiva) equilibrated with 10 mM sodium acetate (pH 4.7) was used to purify the VHHs. The VHH concentration in the stock solution was determined by measuring the absorbance with an Ultrospec 3300 Pro spectrophotometer (Cytiva) at 280 nm.

### 4.3 Antigen binding assay

For surface plasmon resonance (SPR) analysis, the antigen was immobilized on the sensor chip as a ligand, and the VHH sample was injected as an analyte. According to the manufacturer's instructions, F_ab_ from trastuzumab was amine‐coupled to a CM5 sensor chip (Cytiva) at 25°C using 10 μg/mL protein in 20 mM sodium acetate buffer (pH 4.7). Trastuzumab‐F_ab_ proteins in 10 mM sodium acetate (pH 4.7) were immobilized to 1000 resonance units (RU). The dilution series of the analytes was set to 1/2. The analysis was performed on a Biacore X100 instrument (Cytiva) in 10 mM 4‐(2‐hydroxyethyl)‐1‐piperazineethanesulfonic acid (pH 7.4), 150 mM NaCl, 3 mM Ethylenediaminetetraacetic acid, and 0.005% (w/v) P20 surfactant (HBS‐EP, Cytiva) at 20°C. The association reaction was monitored by injecting the sample at various concentrations onto the sensor chip. The dissociation reaction was performed by eluting the bound antigen with HBS‐EP buffer. All experiments were performed at a flow rate of 10 μL/min, association time of 360 s, and dissociation time of 800 s. The sensor chip was regenerated with 10 mM glycine‐HCl buffer (pH 2.5) containing 0.5 M NaCl and equilibrated with HBS‐EP buffer. The capture method was used for clones with incomplete regeneration of the sensor chip by glycine‐HCl buffer. Anti‐Human F_ab_ polyclonal antibodies (Sigma‐Aldrich, 13266) in 10 mM sodium acetate (pH 4.7) were immobilized to 6000 RU. After binding 200 nM Trastuzumab‐F_ab_ for 360 s, the dilution series of VHH protein was set to 1/2. The reference was the value of a channel with no ligand bound, and buffer (HBS‐EP) was used as a blank before the new analyte measurement. Binding kinetics were evaluated using both antigen immobilization and capture formats. The resulting *k*_a_, *k*_d_, and *K*_D_ values were comparable between the two methods (Table S7). For representative clones, the ratios between methods were close to unity (*K*_D_: 0.98 ± 0.45, *k*_a_: 0.77 ± 0.21, *k*_d_: 0.72 ± 0.23).

Sensorgrams were subjected to kinetic analysis using BIAevaluation software (Biacore X100 Evaluation Software, Cytiva). The “1:1 binding model” or “Steady State Affinity analysis” was used to determine dissociation constants.

### 4.4 Protein thermal shift assay

To monitor protein unfolding, fluorescent dyes were used with the Thermal Shift Assay kit (Thermo Fisher Scientific, Rockford, IL, USA). Thermal shift assays were performed using the Step One Plus Real‐Time PCR system (Applied Biosystems): 12.5 μL of 0.2 mg/mL VHH protein, 5 μL of Protein Thermal Shift Buffer, and 2.5 μL × 8 Sypro Orange Protein Stain solution was added to the wells of a 96‐well plate. Water was added to the control samples instead of protein. Samples were heated from 25 to 95°C at a rate of 0.7°C/min. Changes in fluorescence were monitored using ROX filters.

### 4.5 Circular dichroism spectra

Circular dichroism spectra were measured using a J‐820 spectropolarimeter (Jasco) and a 1‐mm cell in PBS (pH 7.4), 10 mM Gly‐HCl (pH 3), 10 mM NaOH (pH 9.5) with 150 mm NaCl, and a protein concentration of 30 μM at 20°C. The CD data were processed using two normalization schemes, depending on the analysis purpose. For VHH antibodies alone, the spectra were expressed as mean residue ellipticity, normalized using protein concentration, path length, and number of amino acid residues. To compare the experimental and calculated spectra of the F_ab_‐VHH complexes, ellipticity was expressed without residue normalization. In this case, the calculated spectra were generated by summing the individual CD spectra of VHH and F_ab_, measured separately under identical conditions.

### 4.6 ELISA

Trastuzumab‐F_ab_ protein (100 μL, 10 μg/mL) in 50 mM carbonic buffer (pH 9.5) was immobilized on Maxisorp 96‐well plates (Thermo Fisher Scientific) and incubated at 4°C overnight. At all stages, the wells were washed 4× with 150 μL PBS containing 0.02% (w/v) Tween 20 (PBS‐T). Unreacted sites on the plastic surfaces were blocked with 3% (v/v) bovine serum albumin (BSA)‐PBS at 25°C for 1 h. Then, 100 μL VHH sample diluted in PBS‐T with 0.1% (v/v) BSA was added to each well. The wells were incubated at 25°C for 2 h, washed four times with PBS‐T and treated with PBS, 0.1 M Gly‐HCL pH 3.0, 0.1 M triethylamine for 15 min at 25°C. After washing with PBS‐T, 100 μL anti‐hishtag antibody was added to each well and incubation continued at 25°C for 1 h. Then, 100 μL of horseradish peroxidase (HRP)‐conjugated anti‐mouse IgG rabbit antibody in PBS‐T containing 0.1% (v/v) BSA was added to each well, and incubation was continued at 25°C for 1 h. Subsequently, 100 μL of 3,3′5,5′‐tetramethylbenzidine (TMB) (SeraCare Life Sciences, Milford, MA, USA) was added to each well and incubated at 25°C for 5 min. The reactions were stopped by adding 100 μL 0.5 M H_2_SO_4_ to each well, and absorbances were measured in a microplate reader (Multiskan FC; Thermo Fisher Scientific) at 450 and 620 nm.

### 4.7 Crystal structure of the VHH‐antigen complex

Trastuzumab‐F_ab_ and L202024 VHH were mixed in equal molar ratios in 20 mM sodium acetate buffer (pH 5.0) and purified using ion exchange column chromatography on a Resource S column (Figure 3b). The analysis revealed the presence of four main peaks, and the relevant samples were then subjected to sodium dodecyl sulfate‐polyacrylamide gel electrophoresis (SDS‐PAGE). Peak A was identified as unreacted F_ab_, while peaks B and C were confirmed as F_ab_‐VHH complexes. However, the presence of F_ab_s with different molecular weights was observed, which is likely attributable to the heterogeneity of F_ab_. Peak D was determined to be unreacted VHH (Figure 3c). Peak C was concentrated to 12.5 mg/mL and crystallization screening was performed using Gryphon to screen crystallization conditions of 384. Under these conditions, Wizard Classic 3 & 4 A8 (20% (w/v) polyethylene glycol 3350, 200 mM potassium nitrate), a polycrystal was obtained. The polycrystal was broken to separate single crystals. x‐ray diffraction data were collected at KEK‐PF AR‐NW12A beam line under nitrogen stream at 100 K using glycerol as cryoprotectant (Table S3). Initial phases were obtained using molecular displacement conducted with the program PHASER in the CCP4 package (McCoy et al., 2007) and experimental models of F_ab_ and VHH from Protein Data Bank (PDB) ID 1N8Z and 5E7B. Subsequent modeling and refinement were carried out with Coot (Emsley et al., 2010) and refmac (Murshudov et al., 1997). The relevant statistics are listed in Table S3. Structural coordinates were deposited to PDB (ID: 9XL8).

### 4.8 Statistical analysis

Statistical analysis was performed with GraphPad Prism 10.4.1 (GraphPad Software, LLC, San Diego, CA, USA).

## AUTHOR CONTRIBUTIONS

The authors confirm their contributions to the article were as follows: **YA‐O** and **YH** designed the study and supervised data collection. **YA‐O** and **YH** prepared **VHH** and analyzed its stability and activity. **TM** performed gene sequence analysis of **VHH** antibodies. **NK**, **NM**, **TF**, and **YI** performed antigen immunization and acquisition of VHH antibody sequences. **MA** analyzed the structure of complex. **YA‐O** and **YH** wrote the article. All authors reviewed the results and approved the final version of the manuscript.

## FUNDING INFORMATION

This study was supported by the Japan Society for the Promotion of Science (JSPS) KAKENHI Grant No. 20K07009 (Yoko Akazawa‐Ogawa, Tomonari Matsuda, and Yoshihisa Hagihara) and JSR Corporation (Yoko Akazawa‐Ogawa, Tomonari Matsuda, Nobuo Miyazaki, Yuji Ito, and Yoshihisa Hagihara).

## CONFLICT OF INTEREST STATEMENT

Author Tetsuo Fukuta is employed by JSR Corporation. Authors Norihiko Kiyose and Nobuo Miyazaki are employed by ARK Resource. Co., Ltd. Kyoto University applied for patents Nos. JP2020177529A. Tomonari Matsuda, Yoko Akazawa‐Ogawa, Nobuo Miyazaki, Tetsuo Fukuta, Yuji Ito, and Yoshihisa Hagihara were listed as inventors. JSR Corporation applied for patents Nos. JP7298607B2. Tomonari Matsuda, Yoko Akazawa‐Ogawa, Nobuo Miyazaki, Norihiko Kiyose, Tetsuo Fukuta, Yuji Ito, and Yoshihisa Hagihara were listed as inventors.

## Supporting information


**Data S1.** These figures and tables present raw data from functional and physicochemical analyses of individual antibodies, as well as supplementary data related to structural analysis.

## Data Availability

The data that support the findings of this study are openly available in PDB at https://www.rcsb.org, reference number 9XL8.
