# Supplementary material for: Optimization of antibody binding by adjusting thermodynamic stability during the maturation of antibody affinities
Source: Protein Sci. 2026 Aug 6;35(9):e70739. doi: 10.1002/pro.70739 (PMC13445567; doi:10.1002/pro.70739)
Supplement: Supplementary file 1 [file PRO-35-e70739-s001.docx]

Supplementary Material

Optimization of antibody binding by adjusting thermodynamic stability during the maturation of antibody affinities

Yoko Akazawa-Ogawa^1^*, Tomonari, Matsuda^2^, Norihiko Kiyose^3^, Nobuo Miyazaki^3^, Tetsuo Fukuta^4^, Motoyasu Adachi^5^, Yuji Ito^6^, Yoshihisa Hagihara^7^*

Email: [yoko-ogawa@aist.go.jp](mailto:yoko-ogawa@aist.go.jp), [hagihara-kappael@aist.go.jp](mailto:hagihara-kappael@aist.go.jp)

This PDF file includes:

Supplementary Figures S1 to S9

Supplementary Tables S1 to S7

**SUPPLEMENTARY FIGURE S1**　 **Phylogenetic sub-clustering of the Ig-15 VHH clones.**

The phylogenetic tree constructed from sequences in the Ig-15 cluster was divided into three subclusters (Subcluster 1–3) based on the branching structure. Clones belonging to each subcluster are indicated in red (Subcluster 1), green (Subcluster 2), and blue (Subcluster 3).

**SUPPLEMENTARY FIGURE S2 CDR3 regions of the Ig-15 cluster**

(A) Alignment of CDR3 sequences. The upstream 4 nucleotides and downstream 3 nucleotides are presumed to be derived from V and J genes, respectively. (B) Phylogenetic tree of CDR3 sequences. The red scale bar indicates the substitution rate (mutation rate) per nucleotide. (C) Alignment of the D-J junction regions.

**SUPPLEMENTARY FIGURE S3 Epitope competition analysis of Ig‑15 cluster VHH antibodies by SPR.**

Trastuzumab F_ab_ was captured using a CM5 sensor chip coated with an anti-F_ab_ polyclonal antibody. Binding of VHH clones (L804 at 100 nM, L7318 at 400 nM, L6805 at 100 nM, L15144 at 400 nM, and L21793 at 400 nM) was evaluated in the presence (red lines) or absence (black lines) of pre‑bound VHH clone L1332 (100 nM). The reduced binding signals observed in the presence of pre-bound L1332 indicate competitive binding to the same or overlapping epitopes.

**SUPPLEMENTARY FIGURE S4 Antigen binding activity of clones in the cluster.**

The affinity of each clone was measured by SPR against immobilized Trastuzumab-F_ab_. The values inside the SPR panels indicate the concentration ranges of VHH clones used as analytes, measured in nM, with a 1:2 dilution series. The “1:1 binding model” was used to determine the *K*_D_. All clones bound to Trastuzumab-F_ab_; however, their affinities varied significantly. L11966, L24189, L61790, L62103, L4899, L15101, L15139, L19817, S1156, L36104, and S1146 were subjected to steady-state affinity analysis based on the maximum RU at a given VHH concentration.

**SUPPLEMENTARY FIGURE S5 Antigen binding activity of clones analyzed for steady-state affinity.**

SPR sensorgrams of clones analyzed by steady-state affinity analysis. The affinity of each clone for trastuzumab-F_ab_ was measured by SPR; the values in the SPR panel indicate the concentration range of the VHH clone used for analysis, measured in nM in a 1:2 dilution series.

**SUPPLEMENTARY FIGURE S6 Correlation analysis between *k*_a_, *k*_d_*,* and bit score.**

Correlations between *k*_a_ or *k*_d_ and the bit score were analyzed using data from clones fitted to the 1:1 binding model (Table S2). The inset in (A) shows an enlarged view of the region enclosed by the dotted line. In (B), the correlation between *k*_d_ and the bit score was analyzed using clones within the dotted-line region after excluding outliers.

**SUPPLEMENTARY FIGURE S7 Thermal unfolding of clones in the Ig-15 cluster.**

VHHs at the concentration of 0.1 mg/ml were subjected to the DSF measurements using Sypro Orange in the buffer of 10 mM Na-phosphate and 10 mM NaCl at pH 7.0. The *T*_m_ value was calculated using the sigmoid fitting of the T half (red line).

**SUPPLEMENTARY FIGURE S8 Assessment of acid and alkali resistance of antibodies and antibody-antigen complexes by CD spectra and ELISA assay.**

(A) pH tolerance of VHH antibody clones that were not completely dissociated at pH 2.5 in the Biacore assay. Clone S3146 was excluded from this analysis due to insufficient protein yield. CD spectra of VHH antibodies (30 µM) were recorded in solutions at pH 3.0, 7.4, and 9.5.

(B) L11966 and L7813, which have been demonstrated to have high binding capacity and stability, and L14600 and S4511, which have been shown to have high binding capacity but low stability, were selected for the purpose of assessing the acid/alkali resistance of the antigen-antibody complex. After the formation of the Trastuzumab-F_ab_ and VHH complexes, solvents with pH 3.0 (blue), 7.4 (black), and 9.5 (red) were added. After washing the dissociated VHH, the remaining VHH antibody amount was measured using polyhistidine tag antibody.

**SUPPLEMENTARY FIGURE S9 The location of somatic mutations.**

Using the L202024 structure, the locations of somatic mutations in each CDR and FR were illustrated using a ballstick model, with the amino acid sequence of L3037 serving as a reference.

Supplementary Table S1 Overview of VHH clones in the Ig-15 cluster

| Clone ID | Annotation / Alias | Sub-cluster | Status | Selection reason | Relative frequency (week) | | | | | | | | | | | | | | | Relative frequency_  sum |
| --- | --- | --- | --- | --- | --- | --- | --- | --- | --- | --- | --- | --- | --- | --- | --- | --- | --- | --- | --- | --- |
|  |  |  |  |  | **Early** | | | | **Middle** | | | | | | **Late** | | | | |  |
|  |  |  |  |  | **1** | **2** | **3** | **4** | **5** | **6** | **7** | **8** | **9** | **10** | **11** | **12** | **13** | **14** | **15** |  |
| S567 |  | **2** | **Selected but excluded** | **Phage display–derived, excluded for low expression** | **0** | **0** | **0** | **0** | **0** | **0** | **0** | **0.003** | **0.19** | **0** | **0** | **0** | **0.055** | **0** | **0** | **0.247** |
| S543 |  | **1** | **Analyzed** | **Phage display–derived** | **0** | **0** | **0** | **0** | **0** | **0** | **0** | **0** | **0** | **0** | **0** | **0** | **0.016** | **0** | **0.212** | **0.228** |
| L847 | **L4105** | **1** | **Not analyzed** | **identical to L4105 in amino acid sequence** | **0** | **0** | **0** | **0** | **0** | **0** | **0** | **0.039** | **0.013** | **0** | **0** | **0** | **0.139** | **0.009** | **0** | **0.201** |
| L804 |  | **1** | **Analyzed** | **clone of subcluster 1 (middle)** | **0** | **0** | **0** | **0** | **0** | **0** | **0** | **0.096** | **0.092** | **0.003** | **0** | **0** | **0** | **0** | **0** | **0.191** |
| L926 | **S2161** | **3** | **Analyzed** | **Phage display–derived** | **0** | **0** | **0** | **0** | **0** | **0** | **0** | **0** | **0** | **0** | **0** | **0.011** | **0.047** | **0.114** | **0.008** | **0.180** |
| S1146 | **L77391, L74886** | **3** | **Analyzed** | **clone of subcluster 3 (middle)** | **0** | **0** | **0** | **0** | **0** | **0** | **0** | **0.021** | **0.043** | **0** | **0** | **0.025** | **0.089** | **0** | **0** | **0.177** |
| S1076 |  | **3** | **Selected but excluded** | **clone of subcluster 3 (middle), excluded for low expression** | **0** | **0** | **0** | **0** | **0** | **0** | **0.005** | **0.111** | **0.040** | **0** | **0** | **0** | **0** | **0** | **0** | **0.157** |
| S877 | **L1332** | **1** | **Not analyzed** | **identical to L1332 in amino acid sequence** | **0** | **0** | **0** | **0** | **0** | **0** | **0** | **0** | **0** | **0** | **0** | **0** | **0** | **0.016** | **0.134** | **0.150** |
| L1463 |  | **3** | **Selected but excluded** | **clone of subcluster 3 (late), excluded for low expression** | **0** | **0** | **0** | **0** | **0** | **0** | **0** | **0** | **0** | **0** | **0** | **0** | **0.095** | **0.036** | **0.008** | **0.138** |
| L1196 | **S5647** | **2** | **Analyzed** | **clone of subcluster 2 (middle)** | **0** | **0** | **0** | **0** | **0** | **0** | **0** | **0.073** | **0.063** | **0** | **0** | **0** | **0** | **0** | **0** | **0.135** |
| S1156 |  | **2** | **Analyzed** | **Phage display–derived** | **0** | **0** | **0** | **0** | **0** | **0** | **0** | **0.101** | **0** | **0** | **0** | **0** | **0** | **0** | **0** | **0.101** |
| L1332 |  | **1** | **Analyzed** | **Phage display–derived** | **0** | **0** | **0** | **0** | **0** | **0** | **0** | **0** | **0** | **0** | **0** | **0** | **0** | **0.087** | **0.012** | **0.099** |
| L1088 |  | **2** | **Analyzed** | **clone of subcluster 2 (middle)** | **0** | **0** | **0** | **0** | **0** | **0** | **0.0008** | **0.078** | **0** | **0** | **0** | **0** | **0** | **0** | **0** | **0.078** |
| S2527 |  | **2** | **Selected but excluded** | **Phage display–derived, excluded for low expression** | **0** | **0** | **0** | **0** | **0.011** | **0.054** | **0.013** | **0** | **0** | **0** | **0** | **0** | **0** | **0** | **0** | **0.078** |
| L3037 |  | **1** | **Analyzed** | **Phage display–derived** | **0** | **0** | **0.04** | **0.037** | **0** | **0** | **0** | **0** | **0** | **0** | **0** | **0** | **0** | **0** | **0** | **0.077** |
| L2157 | **L1463** | **3** | **Selected but excluded** | **identical to L1463 in amino acid sequence** | **0** | **0** | **0** | **0** | **0** | **0** | **0** | **0** | **0** | **0** | **0** | **0** | **0** | **0.061** | **0.003** | **0.064** |
| S1908 |  | **1** | **Analyzed** | **Phage display–derived** | **0** | **0** | **0** | **0** | **0** | **0** | **0** | **0.061** | **0** | **0** | **0** | **0** | **0** | **0** | **0** | **0.061** |
| L2271 |  | **2** | **Selected but excluded** | **clone of subcluster 2 (middle), excluded for low expression** | **0** | **0** | **0** | **0** | **0** | **0** | **0.059** | **0** | **0** | **0** | **0** | **0** | **0** | **0** | **0** | **0.059** |
| L5301 |  | **2** | **Selected but excluded** | **clone of subcluster 2 (late), excluded due to poor solubility** | **0** | **0** | **0** | **0** | **0** | **0** | **0** | **0** | **0** | **0** | **0** | **0** | **0.035** | **0.015** | **0** | **0.049** |
| S2161 | **L926** | **3** | **Not analyzed** | **identical to L926 in amino acid sequence** | **0** | **0** | **0** | **0** | **0** | **0** | **0** | **0** | **0** | **0** | **0** | **0** | **0.047** | **0** | **0** | **0.047** |
| L2362 | **S5267** | **2** | **Analyzed** | **clone of subcluster 2 (middle)** | **0** | **0** | **0** | **0** | **0** | **0** | **0** | **0.044** | **0** | **0** | **0** | **0** | **0** | **0** | **0** | **0.044** |
| L6805 | **S4999** | **2** | **Analyzed** | **Phage display–derived** | **0** | **0** | **0** | **0** | **0** | **0** | **0.002** | **0.019** | **0.006** | **0.009** | **0** | **0** | **0.006** | **0** | **0** | **0.042** |
| L4899 |  | **3** | **Analyzed** | **clone of subcluster 3 (middle)** | **0** | **0** | **0** | **0** | **0** | **0** | **0** | **0.024** | **0.018** | **0** | **0** | **0** | **0** | **0** | **0** | **0.042** |
| L4105 | **L847** | **1** | **Analyzed** | **Phage display–derived** | **0** | **0** | **0** | **0** | **0** | **0** | **0** | **0.009** | **0.032** | **0.0005** | **0** | **0** | **0** | **0** | **0** | **0.042** |
| S3449 |  | **1** | **Analyzed** | **clone of subcluster 1 (late)** | **0** | **0** | **0** | **0** | **0** | **0** | **0** | **0** | **0** | **0** | **0** | **0** | **0.009** | **0** | **0.032** | **0.041** |
| L3763 |  | **2** | **Selected but excluded** | **clone of subcluster 2 (early), excluded due to poor solubility** | **0** | **0** | **0** | **0.040** | **0** | **0** | **0** | **0** | **0** | **0** | **0** | **0** | **0** | **0** | **0** | **0.040** |
| S4618 | **S12210** | **1** | **Selected but excluded** | **clone of subcluster 1 (late), excluded for low expression** | **0** | **0** | **0** | **0** | **0** | **0** | **0** | **0** | **0** | **0** | **0** | **0** | **0.013** | **0.023** | **0** | **0.036** |
| S3146 |  | **1** | **Analyzed** | **clone of subcluster 1 (late)** | **0** | **0** | **0** | **0** | **0** | **0** | **0** | **0** | **0** | **0** | **0** | **0** | **0.031** | **0.005** | **0** | **0.036** |
| L3585 | **S8671** | **1** | **Analyzed** | **clone of subcluster 1 (middle)** | **0** | **0** | **0** | **0** | **0** | **0** | **0** | **0.031** | **0** | **0** | **0** | **0** | **0** | **0** | **0** | **0.031** |
| S3143 | **L87697** | **3** | **Selected but excluded** | **clone of subcluster 3 (late), excluded for low expression** | **0** | **0** | **0** | **0** | **0** | **0** | **0** | **0** | **0** | **0** | **0** | **0** | **0.031** | **0** | **0** | **0.031** |
| L21793 |  | **1** | **Analyzed** | **clone of subcluster 1 (middle)** | **0** | **0** | **0** | **0** | **0.013** | **0.007** | **0.010** | **0** | **0** | **0** | **0** | **0** | **0** | **0** | **0** | **0.029** |
| S4371 |  | **2** | **Not analyzed** | **not selected for detailed analysis** | **0** | **0** | **0** | **0** | **0** | **0.028** | **0** | **0** | **0** | **0** | **0** | **0** | **0** | **0** | **0** | **0.028** |
| L7813 |  | **2** | **Analyzed** | **Phage display–derived** | **0** | **0** | **0** | **0** | **0** | **0** | **0** | **0** | **0** | **0** | **0** | **0** | **0.025** | **0** | **0** | **0.025** |
| L15101 |  | **3** | **Analyzed** | **clone of subcluster 3 (middle)** | **0** | **0** | **0** | **0** | **0.013** | **0.012** | **0** | **0** | **0** | **0** | **0** | **0** | **0** | **0** | **0** | **0.025** |
| L19817 |  | **3** | **Analyzed** | **clone of subcluster 3 (middle)** | **0** | **0** | **0** | **0** | **0** | **0.010** | **0.009** | **0.004** | **0** | **0** | **0** | **0** | **0** | **0** | **0** | **0.023** |
| L7318 |  | **2** | **Analyzed** | **clone of subcluster 2 (early)** | **0** | **0** | **0** | **0.023** | **0** | **0** | **0** | **0** | **0** | **0** | **0** | **0** | **0** | **0** | **0** | **0.023** |
| S4008 |  | **1** | **Analyzed** | **clone of subcluster 1 (late)** | **0** | **0** | **0** | **0** | **0** | **0** | **0** | **0** | **0** | **0** | **0** | **0** | **0.023** | **0** | **0** | **0.023** |
| L5236 |  | **2** | **Not analyzed** | **not selected for detailed analysis** | **0** | **0** | **0** | **0** | **0** | **0** | **0** | **0.023** | **0** | **0** | **0** | **0** | **0** | **0** | **0** | **0.023** |
| S4700 |  | **1** | **Not analyzed** | **not selected for detailed analysis** | **0** | **0** | **0** | **0** | **0** | **0** | **0** | **0.022** | **0** | **0** | **0** | **0** | **0** | **0** | **0** | **0.022** |
| S5258 |  | **2** | **Not analyzed** | **not selected for detailed analysis** | **0** | **0** | **0** | **0** | **0** | **0** | **0.021** | **0** | **0** | **0** | **0** | **0** | **0** | **0** | **0** | **0.021** |
| L9347 |  | **2** | **Selected but excluded** | **clone of subcluster 2 (early), excluded for low expression** | **0** | **0** | **0** | **0.019** | **0.001** | **0** | **0** | **0** | **0** | **0** | **0** | **0** | **0** | **0** | **0** | **0.020** |
| S4511 |  | **1** | **Analyzed** | **representative clone of subcluster 1 (late)** | **0** | **0** | **0** | **0** | **0** | **0** | **0** | **0** | **0** | **0** | **0** | **0** | **0.020** | **0** | **0** | **0.020** |
| S5267 | **L2362** | **2** | **Not analyzed** | **identical to L2362 in amino acid sequence** | **0** | **0** | **0** | **0** | **0** | **0** | **0** | **0.019** | **0** | **0** | **0** | **0** | **0** | **0** | **0** | **0.019** |
| S4727 |  | **1** | **Analyzed** | **Phage display–derived** | **0** | **0** | **0** | **0** | **0** | **0** | **0** | **0** | **0** | **0** | **0** | **0** | **0.019** | **0** | **0** | **0.019** |
| S5754 |  | **2** | **Not analyzed** | **not selected for detailed analysis** | **0** | **0** | **0** | **0** | **0** | **0** | **0.018** | **0** | **0** | **0** | **0** | **0** | **0** | **0** | **0** | **0.018** |
| S4999 | **L6805** | **2** | **Not analyzed** | **identical to L6805 in amino acid sequence** | **0** | **0** | **0** | **0** | **0** | **0** | **0** | **0** | **0** | **0** | **0** | **0** | **0.018** | **0** | **0** | **0.018** |
| L27341 |  | **2** | **Not analyzed** | **not selected for detailed analysis** | **0** | **0** | **0** | **0** | **0** | **0** | **0.008** | **0** | **0** | **0.001** | **0** | **0** | **0.008** | **0** | **0** | **0.018** |
| L12981 |  | **1** | **Not analyzed** | **not selected for detailed analysis** | **0** | **0** | **0** | **0** | **0** | **0** | **0** | **0.0006** | **0** | **0** | **0** | **0** | **0.017** | **0** | **0** | **0.017** |
| S5141 | **S8100, S9841** | **1** | **Not analyzed** | **not selected for detailed analysis** | **0** | **0** | **0** | **0** | **0** | **0** | **0** | **0** | **0** | **0** | **0** | **0** | **0.017** | **0** | **0** | **0.017** |
| S5293 |  | **1** | **Not analyzed** | **not selected for detailed analysis** | **0** | **0** | **0** | **0** | **0** | **0** | **0** | **0** | **0** | **0** | **0** | **0** | **0.017** | **0** | **0** | **0.017** |
| S5942 |  | **1** | **Analyzed** | **clone of subcluster 1 (middle)** | **0** | **0** | **0** | **0** | **0** | **0** | **0** | **0.017** | **0** | **0** | **0** | **0** | **0** | **0** | **0** | **0.017** |
| S6689 |  | **3** | **Not analyzed** | **not selected for detailed analysis** | **0** | **0** | **0** | **0** | **0** | **0.016** | **0** | **0** | **0** | **0** | **0** | **0** | **0** | **0** | **0** | **0.016** |
| L11966 |  | **3** | **Analyzed** | **clone of subcluster 3 (early)** | **0** | **0** | **0** | **0.016** | **0** | **0** | **0** | **0** | **0** | **0** | **0** | **0** | **0** | **0** | **0** | **0.016** |
| S5647 | **L1196** | **2** | **Not analyzed** | **identical to L1196 in amino acid sequence** | **0** | **0** | **0** | **0** | **0** | **0** | **0** | **0** | **0** | **0** | **0** | **0** | **0.016** | **0** | **0** | **0.016** |
| L24387 |  | **3** | **Selected but excluded** | **clone of subcluster 3 (middle), excluded for low expression** | **0** | **0** | **0** | **0** | **0** | **0.008** | **0.0041** | **0.003** | **0** | **0** | **0** | **0** | **0** | **0** | **0** | **0.016** |
| L16846 |  | **1** | **Not analyzed** | **not selected for detailed analysis** | **0** | **0** | **0** | **0** | **0** | **0** | **0** | **0** | **0** | **0** | **0.0006** | **0** | **0.014** | **0.001** | **0** | **0.015** |
| S6704 |  | **1** | **Not analyzed** | **not selected for detailed analysis** | **0** | **0** | **0** | **0** | **0** | **0** | **0.015** | **0** | **0** | **0** | **0** | **0** | **0** | **0** | **0** | **0.015** |
| S5980 |  | **1** | **Not analyzed** | **not selected for detailed analysis** | **0** | **0** | **0** | **0** | **0** | **0** | **0** | **0** | **0** | **0** | **0** | **0** | **0.014** | **0** | **0** | **0.014** |
| L17082 |  | **1** | **Not analyzed** | **not selected for detailed analysis** | **0** | **0** | **0** | **0** | **0** | **0** | **0** | **0** | **0** | **0** | **0** | **0** | **0.014** | **0** | **0** | **0.014** |
| L10024 |  | **3** | **Not analyzed** | **not selected for detailed analysis** | **0** | **0** | **0** | **0** | **0** | **0** | **0** | **0.014** | **0** | **0** | **0** | **0** | **0** | **0** | **0** | **0.014** |
| L12866 |  | **3** | **Not analyzed** | **not selected for detailed analysis** | **0** | **0** | **0** | **0** | **0** | **0.014** | **0** | **0** | **0** | **0** | **0** | **0** | **0** | **0** | **0** | **0.014** |
| L_36104 |  | **3** | **Analyzed** | **Phage display–derived** | **0** | **0** | **0** | **0** | **0** | **0** | **0** | **0** | **0** | **0** | **0** | **0** | **0.007** | **0.006** | **0** | **0.013** |
| L_10624 |  | **2** | **Not analyzed** | **not selected for detailed analysis** | **0** | **0** | **0** | **0** | **0** | **0** | **0** | **0.013** | **0** | **0** | **0** | **0** | **0** | **0** | **0** | **0.013** |
| L_10632 |  | **2** | **Not analyzed** | **not selected for detailed analysis** | **0** | **0** | **0** | **0** | **0** | **0** | **0** | **0.013** | **0** | **0** | **0** | **0** | **0** | **0** | **0** | **0.013** |
| S7655 |  | **1** | **Not analyzed** | **not selected for detailed analysis** | **0** | **0** | **0** | **0** | **0** | **0.013** | **0** | **0** | **0** | **0** | **0** | **0** | **0** | **0** | **0** | **0.013** |
| L15144 |  | **3** | **Analyzed** | **clone of subcluster 3 (middle)** | **0** | **0** | **0** | **0** | **0** | **0.012** | **0** | **0.0006** | **0** | **0** | **0** | **0** | **0** | **0** | **0** | **0.013** |
| S6792 |  | **1** | **Not analyzed** | **not selected for detailed analysis** | **0** | **0** | **0** | **0** | **0** | **0** | **0** | **0** | **0** | **0** | **0** | **0** | **0.012** | **0** | **0** | **0.012** |
| L15139 |  | **1** | **Analyzed** | **clone of subcluster 1 (middle)** | **0** | **0** | **0** | **0** | **0** | **0.012** | **0** | **0** | **0** | **0** | **0** | **0** | **0** | **0** | **0** | **0.012** |
| L12076 |  | **1** | **Not analyzed** | **not selected for detailed analysis** | **0** | **0** | **0** | **0** | **0** | **0** | **0** | **0.012** | **0** | **0** | **0** | **0** | **0** | **0** | **0** | **0.012** |
| S8217 |  | **2** | **Not analyzed** | **not selected for detailed analysis** | **0** | **0** | **0** | **0** | **0** | **0.012** | **0** | **0** | **0** | **0** | **0** | **0** | **0** | **0** | **0** | **0.012** |
| S7793 |  | **1** | **Not analyzed** | **not selected for detailed analysis** | **0** | **0** | **0** | **0** | **0** | **0** | **0** | **0** | **0** | **0** | **0** | **0** | **0** | **0.012** | **0** | **0.012** |
| L18834 |  | **3** | **Selected but excluded** | **clone of subcluster 3 (late), excluded due to poor solubility** | **0** | **0** | **0** | **0** | **0** | **0** | **0** | **0** | **0** | **0** | **0** | **0** | **0** | **0.011** | **0** | **0.011** |
| L30775 |  | **1** | **Not analyzed** | **not selected for detailed analysis** | **0** | **0** | **0** | **0** | **0** | **0** | **0** | **0.006** | **0** | **0** | **0** | **0** | **0.006** | **0** | **0** | **0.011** |
| S7104 |  | **3** | **Not analyzed** | **not selected for detailed analysis** | **0** | **0** | **0** | **0** | **0.011** | **0** | **0** | **0** | **0** | **0** | **0** | **0** | **0** | **0** | **0** | **0.011** |
| S8100 | **S_5141** | **1** | **Not analyzed** | **not selected for detailed analysis** | **0** | **0** | **0** | **0** | **0** | **0** | **0** | **0** | **0** | **0** | **0** | **0** | **0** | **0.011** | **0** | **0.011** |
| L13279 |  | **1** | **Not analyzed** | **not selected for detailed analysis** | **0** | **0** | **0** | **0** | **0** | **0** | **0** | **0** | **0** | **0** | **0** | **0.011** | **0** | **0** | **0** | **0.011** |
| L14053 |  | **1** | **Not analyzed** | **not selected for detailed analysis** | **0** | **0** | **0** | **0** | **0** | **0** | **0** | **0.011** | **0** | **0** | **0** | **0** | **0** | **0** | **0** | **0.011** |
| L39174 |  | **3** | **Not analyzed** | **not selected for detailed analysis** | **0** | **0** | **0** | **0** | **0.010** | **0** | **0** | **0** | **0** | **0** | **0** | **0** | **0** | **0** | **0** | **0.010** |
| L23014 |  | **3** | **Selected but excluded** | **clone of subcluster 3 (late), excluded due to poor solubility** | **0** | **0** | **0** | **0** | **0** | **0** | **0** | **0** | **0** | **0** | **0** | **0** | **0** | **0.010** | **0** | **0.010** |
| L14600 |  | **1** | **Analyzed** | **clone of subcluster 1 (late)** | **0** | **0** | **0** | **0** | **0** | **0** | **0** | **0** | **0** | **0** | **0** | **0** | **0** | **0** | **0.009** | **0.009** |
| S8671 |  | **1** | **Not analyzed** | **not selected for detailed analysis** | **0** | **0** | **0** | **0** | **0** | **0** | **0** | **0.009** | **0** | **0** | **0** | **0** | **0** | **0** | **0** | **0.009** |
| S9262 |  | **3** | **Not analyzed** | **not selected for detailed analysis** | **0** | **0** | **0** | **0** | **0** | **0.009** | **0** | **0** | **0** | **0** | **0** | **0** | **0** | **0** | **0** | **0.009** |
| S9293 |  | **1** | **Not analyzed** | **not selected for detailed analysis** | **0** | **0** | **0** | **0** | **0** | **0.009** | **0** | **0** | **0** | **0** | **0** | **0** | **0** | **0** | **0** | **0.009** |
| L24189 |  | **1** | **Analyzed** | **clone of subcluster 1 (early)** | **0** | **0** | **0** | **0.009** | **0** | **0** | **0** | **0** | **0** | **0** | **0** | **0** | **0** | **0** | **0** | **0.009** |
| S9641 |  | **3** | **Not analyzed** | **not selected for detailed analysis** | **0** | **0** | **0** | **0** | **0** | **0.008** | **0** | **0** | **0** | **0** | **0** | **0** | **0** | **0** | **0** | **0.008** |
| L31740 |  | **1** | **Not analyzed** | **not selected for detailed analysis** | **0** | **0** | **0** | **0** | **0** | **0** | **0** | **0** | **0** | **0** | **0** | **0** | **0.008** | **0** | **0** | **0.008** |
| L24394 |  | **3** | **Not analyzed** | **not selected for detailed analysis** | **0** | **0** | **0** | **0** | **0** | **0.008** | **0** | **0** | **0** | **0** | **0** | **0** | **0** | **0** | **0** | **0.008** |
| L26958 |  | **1** | **Not analyzed** | **not selected for detailed analysis** | **0** | **0** | **0** | **0.008** | **0** | **0** | **0** | **0** | **0** | **0** | **0** | **0** | **0** | **0** | **0** | **0.008** |
| S9507 |  | **1** | **Not analyzed** | **not selected for detailed analysis** | **0** | **0** | **0** | **0** | **0** | **0** | **0** | **0** | **0** | **0** | **0** | **0** | **0** | **0.008** | **0** | **0.008** |
| S9373 |  | **3** | **Not analyzed** | **not selected for detailed analysis** | **0** | **0** | **0** | **0** | **0** | **0** | **0** | **0.008** | **0** | **0** | **0** | **0** | **0** | **0** | **0** | **0.008** |
| S9385 |  | **1** | **Not analyzed** | **not selected for detailed analysis** | **0** | **0** | **0** | **0** | **0** | **0** | **0** | **0.008** | **0** | **0** | **0** | **0** | **0** | **0** | **0** | **0.008** |
| L27421 |  | **2** | **Not analyzed** | **not selected for detailed analysis** | **0** | **0** | **0** | **0** | **0** | **0** | **0.0008** | **0** | **0.007** | **0** | **0** | **0** | **0** | **0** | **0** | **0.008** |
| L52914 |  | **3** | **Not analyzed** | **not selected for detailed analysis** | **0** | **0** | **0** | **0** | **0.0071** | **0.0008** | **0** | **0** | **0** | **0** | **0** | **0** | **0** | **0** | **0** | **0.008** |
| S9965 |  | **2** | **Not analyzed** | **not selected for detailed analysis** | **0** | **0** | **0** | **0** | **0** | **0.008** | **0** | **0** | **0** | **0** | **0** | **0** | **0** | **0** | **0** | **0.008** |
| L36103 |  | **3** | **Not analyzed** | **not selected for detailed analysis** | **0** | **0** | **0** | **0** | **0** | **0** | **0** | **0** | **0** | **0** | **0** | **0** | **0.007** | **0** | **0** | **0.007** |
| S9841 | **S8100, S5141** | **1** | **Not analyzed** | **not selected for detailed analysis** | **0** | **0** | **0** | **0** | **0** | **0** | **0** | **0** | **0** | **0** | **0** | **0** | **0** | **0.007** | **0** | **0.007** |
| L45640 |  | **2** | **Not analyzed** | **not selected for detailed analysis** | **0** | **0** | **0** | **0** | **0** | **0.0008** | **0** | **0.004** | **0.003** | **0** | **0** | **0** | **0** | **0** | **0** | **0.007** |
| L62795 |  | **2** | **Not analyzed** | **not selected for detailed analysis** | **0** | **0** | **0** | **0** | **0.004** | **0.003** | **0** | **0** | **0** | **0** | **0** | **0** | **0** | **0** | **0** | **0.007** |
| L40381 |  | **1** | **Not analyzed** | **not selected for detailed analysis** | **0** | **0** | **0** | **0** | **0** | **0** | **0** | **0** | **0.001** | **0** | **0** | **0** | **0.007** | **0** | **0** | **0.007** |
| L30422 |  | **3** | **Not analyzed** | **not selected for detailed analysis** | **0** | **0** | **0** | **0** | **0** | **0.007** | **0** | **0** | **0** | **0** | **0** | **0** | **0** | **0** | **0** | **0.007** |
| L30466 |  | **2** | **Not analyzed** | **not selected for detailed analysis** | **0** | **0** | **0** | **0** | **0** | **0.007** | **0** | **0** | **0** | **0** | **0** | **0** | **0** | **0** | **0** | **0.007** |
| L30482 |  | **2** | **Not analyzed** | **not selected for detailed analysis** | **0** | **0** | **0** | **0** | **0** | **0.007** | **0** | **0** | **0** | **0** | **0** | **0** | **0** | **0** | **0** | **0.007** |
| L30500 |  | **2** | **Not analyzed** | **not selected for detailed analysis** | **0** | **0** | **0** | **0** | **0** | **0.007** | **0** | **0** | **0** | **0** | **0** | **0** | **0** | **0** | **0** | **0.007** |
| L34741 |  | **2** | **Not analyzed** | **not selected for detailed analysis** | **0** | **0** | **0** | **0** | **0** | **0** | **0.007** | **0** | **0** | **0** | **0** | **0** | **0** | **0** | **0** | **0.007** |
| L47210 |  | **3** | **Not analyzed** | **not selected for detailed analysis** | **0** | **0** | **0** | **0** | **0** | **0** | **0** | **0** | **0** | **0.0005** | **0** | **0** | **0.006** | **0** | **0** | **0.006** |
| S10790 |  | **1** | **Not analyzed** | **not selected for detailed analysis** | **0** | **0** | **0** | **0** | **0** | **0** | **0** | **0** | **0** | **0** | **0** | **0** | **0** | **0.006** | **0** | **0.006** |
| L34440 |  | **1** | **Not analyzed** | **not selected for detailed analysis** | **0** | **0** | **0** | **0** | **0** | **0.006** | **0** | **0** | **0** | **0** | **0** | **0** | **0** | **0** | **0** | **0.006** |
| L34513 |  | **3** | **Not analyzed** | **not selected for detailed analysis** | **0** | **0** | **0** | **0** | **0** | **0.006** | **0** | **0** | **0** | **0** | **0** | **0** | **0** | **0** | **0** | **0.006** |
| L39471 |  | **2** | **Not analyzed** | **not selected for detailed analysis** | **0** | **0** | **0** | **0** | **0** | **0.005** | **0** | **0.001** | **0** | **0** | **0** | **0** | **0** | **0** | **0** | **0.006** |
| L30802 |  | **1** | **Not analyzed** | **not selected for detailed analysis** | **0** | **0** | **0** | **0** | **0** | **0** | **0** | **0.006** | **0** | **0** | **0** | **0** | **0** | **0** | **0** | **0.006** |
| L54193 |  | **1** | **Not analyzed** | **not selected for detailed analysis** | **0** | **0** | **0** | **0** | **0** | **0** | **0** | **0.002** | **0.004** | **0** | **0** | **0** | **0** | **0** | **0** | **0.005** |
| S11457 |  | **1** | **Not analyzed** | **not selected for detailed analysis** | **0** | **0** | **0** | **0** | **0** | **0.005** | **0** | **0** | **0** | **0** | **0** | **0** | **0** | **0** | **0** | **0.005** |
| S11698 |  | **2** | **Not analyzed** | **not selected for detailed analysis** | **0** | **0** | **0** | **0** | **0** | **0** | **0** | **0** | **0** | **0** | **0.005** | **0** | **0** | **0** | **0** | **0.005** |
| L39389 |  | **2** | **Not analyzed** | **not selected for detailed analysis** | **0** | **0** | **0** | **0** | **0** | **0.005** | **0** | **0** | **0** | **0** | **0** | **0** | **0** | **0** | **0** | **0.005** |
| L39390 |  | **3** | **Not analyzed** | **not selected for detailed analysis** | **0** | **0** | **0** | **0** | **0** | **0.005** | **0** | **0** | **0** | **0** | **0** | **0** | **0** | **0** | **0** | **0.005** |
| L39815 |  | **2** | **Not analyzed** | **not selected for detailed analysis** | **0** | **0** | **0** | **0** | **0** | **0** | **0** | **0.004** | **0.001** | **0** | **0** | **0** | **0** | **0** | **0** | **0.005** |
| L34884 |  | **3** | **Not analyzed** | **not selected for detailed analysis** | **0** | **0** | **0** | **0** | **0** | **0** | **0** | **0.005** | **0** | **0** | **0** | **0** | **0** | **0** | **0** | **0.005** |
| L34985 |  | **2** | **Not analyzed** | **not selected for detailed analysis** | **0** | **0** | **0** | **0** | **0** | **0** | **0** | **0.005** | **0** | **0** | **0** | **0** | **0** | **0** | **0** | **0.005** |
| L45041 |  | **2** | **Not analyzed** | **not selected for detailed analysis** | **0** | **0** | **0** | **0.005** | **0** | **0** | **0** | **0** | **0** | **0** | **0** | **0** | **0** | **0** | **0** | **0.005** |
| L77985 |  | **3** | **Not analyzed** | **not selected for detailed analysis** | **0** | **0** | **0** | **0** | **0** | **0** | **0** | **0** | **0.002** | **0** | **0** | **0** | **0.003** | **0** | **0** | **0.005** |
| L35748 |  | **3** | **Not analyzed** | **not selected for detailed analysis** | **0** | **0** | **0** | **0** | **0** | **0** | **0** | **0** | **0** | **0** | **0.005** | **0** | **0** | **0** | **0** | **0.005** |
| L45418 |  | **2** | **Not analyzed** | **not selected for detailed analysis** | **0** | **0** | **0** | **0** | **0** | **0.005** | **0** | **0** | **0** | **0** | **0** | **0** | **0** | **0** | **0** | **0.005** |
| L45466 |  | **3** | **Not analyzed** | **not selected for detailed analysis** | **0** | **0** | **0** | **0** | **0** | **0.005** | **0** | **0** | **0** | **0** | **0** | **0** | **0** | **0** | **0** | **0.005** |
| L45467 |  | **3** | **Not analyzed** | **not selected for detailed analysis** | **0** | **0** | **0** | **0** | **0** | **0.005** | **0** | **0** | **0** | **0** | **0** | **0** | **0** | **0** | **0** | **0.005** |
| L45604 |  | **3** | **Not analyzed** | **not selected for detailed analysis** | **0** | **0** | **0** | **0** | **0** | **0.005** | **0** | **0** | **0** | **0** | **0** | **0** | **0** | **0** | **0** | **0.005** |
| L45605 |  | **2** | **Not analyzed** | **not selected for detailed analysis** | **0** | **0** | **0** | **0** | **0** | **0.005** | **0** | **0** | **0** | **0** | **0** | **0** | **0** | **0** | **0** | **0.005** |
| L87180 |  | **3** | **Not analyzed** | **not selected for detailed analysis** | **0** | **0** | **0** | **0** | **0.003** | **0.002** | **0** | **0** | **0** | **0** | **0** | **0** | **0** | **0** | **0** | **0.004** |
| S11959 |  | **1** | **Not analyzed** | **not selected for detailed analysis** | **0** | **0** | **0** | **0** | **0** | **0** | **0.004** | **0** | **0** | **0** | **0** | **0** | **0** | **0** | **0** | **0.004** |
| L46542 |  | **1** | **Not analyzed** | **not selected for detailed analysis** | **0** | **0** | **0** | **0** | **0** | **0** | **0** | **0** | **0.004** | **0** | **0** | **0** | **0** | **0** | **0** | **0.004** |
| L46543 |  | **1** | **Not analyzed** | **not selected for detailed analysis** | **0** | **0** | **0** | **0** | **0** | **0** | **0** | **0** | **0.004** | **0** | **0** | **0** | **0** | **0** | **0** | **0.004** |
| L46692 |  | **2** | **Not analyzed** | **not selected for detailed analysis** | **0** | **0** | **0** | **0** | **0** | **0** | **0** | **0** | **0.004** | **0** | **0** | **0** | **0** | **0** | **0** | **0.004** |
| L74886 |  | **2** | **Not analyzed** | **not selected for detailed analysis** | **0** | **0** | **0** | **0** | **0.004** | **0** | **0** | **0** | **0** | **0** | **0** | **0** | **0** | **0** | **0** | **0.004** |
| L52367 |  | **2** | **Not analyzed** | **not selected for detailed analysis** | **0** | **0** | **0** | **0.004** | **0** | **0** | **0** | **0** | **0** | **0** | **0** | **0** | **0** | **0** | **0** | **0.004** |
| L53503 |  | **3** | **Not analyzed** | **not selected for detailed analysis** | **0** | **0** | **0** | **0** | **0** | **0** | **0.004** | **0** | **0** | **0** | **0** | **0** | **0** | **0** | **0** | **0.004** |
| L53672 |  | **1** | **Not analyzed** | **not selected for detailed analysis** | **0** | **0** | **0** | **0** | **0** | **0** | **0.004** | **0** | **0** | **0** | **0** | **0** | **0** | **0** | **0** | **0.004** |
| S12210 | **S4618** | **1** | **Not analyzed** | **identical in amino acid sequence to S4618** | **0** | **0** | **0** | **0** | **0** | **0** | **0** | **0** | **0** | **0** | **0** | **0** | **0** | **0.004** | **0** | **0.004** |
| S12015 |  | **1** | **Not analyzed** | **not selected for detailed analysis** | **0** | **0** | **0** | **0** | **0** | **0** | **0** | **0.004** | **0** | **0** | **0** | **0** | **0** | **0** | **0** | **0.004** |
| L53123 |  | **2** | **Not analyzed** | **not selected for detailed analysis** | **0** | **0** | **0** | **0** | **0** | **0.004** | **0** | **0** | **0** | **0** | **0** | **0** | **0** | **0** | **0** | **0.004** |
| L53229 |  | **2** | **Not analyzed** | **not selected for detailed analysis** | **0** | **0** | **0** | **0** | **0** | **0.004** | **0** | **0** | **0** | **0** | **0** | **0** | **0** | **0** | **0** | **0.004** |
| L46161 |  | **3** | **Not analyzed** | **not selected for detailed analysis** | **0** | **0** | **0** | **0** | **0** | **0** | **0** | **0.004** | **0** | **0** | **0** | **0** | **0** | **0** | **0** | **0.004** |
| L46335 |  | **1** | **Not analyzed** | **not selected for detailed analysis** | **0** | **0** | **0** | **0** | **0** | **0** | **0** | **0.004** | **0** | **0** | **0** | **0** | **0** | **0** | **0** | **0.004** |
| L47276 |  | **1** | **Not analyzed** | **not selected for detailed analysis** | **0** | **0** | **0** | **0** | **0** | **0** | **0** | **0** | **0** | **0** | **0.004** | **0** | **0** | **0** | **0** | **0.004** |
| L61790 |  | **3** | **Analyzed** | **clone of subcluster 3 (early)** | **0** | **0** | **0** | **0.003** | **0** | **0** | **0** | **0** | **0** | **0** | **0** | **0** | **0** | **0** | **0** | **0.003** |
| L62103 |  | **2** | **Analyzed** | **clone of subcluster 2 (early)** | **0** | **0** | **0** | **0.003** | **0** | **0** | **0** | **0** | **0** | **0** | **0** | **0** | **0** | **0** | **0** | **0.003** |
| L77059 |  | **1** | **Not analyzed** | **not selected for detailed analysis** | **0** | **0** | **0** | **0** | **0** | **0** | **0** | **0.002** | **0.001** | **0** | **0** | **0** | **0** | **0** | **0** | **0.003** |
| L54031 |  | **2** | **Not analyzed** | **not selected for detailed analysis** | **0** | **0** | **0** | **0** | **0** | **0** | **0** | **0.003** | **0** | **0** | **0** | **0** | **0** | **0** | **0** | **0.003** |
| L63248 |  | **1** | **Not analyzed** | **not selected for detailed analysis** | **0** | **0** | **0** | **0** | **0** | **0.003** | **0** | **0** | **0** | **0** | **0** | **0** | **0** | **0** | **0** | **0.003** |
| L63249 |  | **2** | **Not analyzed** | **not selected for detailed analysis** | **0** | **0** | **0** | **0** | **0** | **0.003** | **0** | **0** | **0** | **0** | **0** | **0** | **0** | **0** | **0** | **0.003** |
| L64950 |  | **1** | **Not analyzed** | **not selected for detailed analysis** | **0** | **0** | **0** | **0** | **0** | **0** | **0** | **0** | **0.003** | **0** | **0** | **0** | **0** | **0** | **0** | **0.003** |
| L65328 |  | **3** | **Not analyzed** | **not selected for detailed analysis** | **0** | **0** | **0** | **0** | **0** | **0** | **0** | **0** | **0.003** | **0** | **0** | **0** | **0** | **0** | **0** | **0.003** |
| L65330 |  | **3** | **Not analyzed** | **not selected for detailed analysis** | **0** | **0** | **0** | **0** | **0** | **0** | **0** | **0** | **0.003** | **0** | **0** | **0** | **0** | **0** | **0** | **0.003** |
| L189828 |  | **1** | **Not analyzed** | **not selected for detailed analysis** | **0** | **0** | **0** | **0** | **0.003** | **0** | **0** | **0** | **0** | **0** | **0** | **0** | **0** | **0** | **0** | **0.003** |
| L79900 |  | **3** | **Not analyzed** | **not selected for detailed analysis** | **0** | **0** | **0** | **0** | **0** | **0** | **0** | **0** | **0** | **0** | **0** | **0** | **0.003** | **0** | **0** | **0.003** |
| L64479 |  | **2** | **Not analyzed** | **not selected for detailed analysis** | **0** | **0** | **0** | **0** | **0** | **0** | **0** | **0.003** | **0** | **0** | **0** | **0** | **0** | **0** | **0** | **0.003** |
| L75765 |  | **1** | **Not analyzed** | **not selected for detailed analysis** | **0** | **0** | **0** | **0** | **0** | **0** | **0.002** | **0** | **0** | **0** | **0** | **0** | **0** | **0** | **0** | **0.002** |
| L66465 |  | **1** | **Not analyzed** | **not selected for detailed analysis** | **0** | **0** | **0** | **0** | **0** | **0** | **0** | **0** | **0** | **0** | **0.002** | **0** | **0** | **0** | **0** | **0.002** |
| L66760 | **S1146, L77391** | **3** | **Not analyzed** | **identical in amino acid sequence to S1146** | **0** | **0** | **0** | **0** | **0** | **0** | **0** | **0** | **0** | **0** | **0.002** | **0** | **0** | **0** | **0** | **0.002** |
| L75089 |  | **1** | **Not analyzed** | **not selected for detailed analysis** | **0** | **0** | **0** | **0** | **0** | **0.002** | **0** | **0** | **0** | **0** | **0** | **0** | **0** | **0** | **0** | **0.002** |
| L75450 |  | **2** | **Not analyzed** | **not selected for detailed analysis** | **0** | **0** | **0** | **0** | **0** | **0.002** | **0** | **0** | **0** | **0** | **0** | **0** | **0** | **0** | **0** | **0.002** |
| L75451 |  | **1** | **Not analyzed** | **not selected for detailed analysis** | **0** | **0** | **0** | **0** | **0** | **0.002** | **0** | **0** | **0** | **0** | **0** | **0** | **0** | **0** | **0** | **0.002** |
| L75452 |  | **2** | **Not analyzed** | **not selected for detailed analysis** | **0** | **0** | **0** | **0** | **0** | **0.002** | **0** | **0** | **0** | **0** | **0** | **0** | **0** | **0** | **0** | **0.002** |
| L75453 |  | **1** | **Not analyzed** | **not selected for detailed analysis** | **0** | **0** | **0** | **0** | **0** | **0.002** | **0** | **0** | **0** | **0** | **0** | **0** | **0** | **0** | **0** | **0.002** |
| L77391 | **S1146, L66760** | **3** | **Not analyzed** | **identical in amino acid sequence to S1146** | **0** | **0** | **0** | **0** | **0** | **0** | **0** | **0** | **0.002** | **0** | **0** | **0** | **0** | **0** | **0** | **0.002** |
| S13330 |  | **1** | **Not analyzed** | **not selected for detailed analysis** | **0** | **0** | **0** | **0** | **0** | **0** | **0** | **0** | **0** | **0** | **0** | **0** | **0** | **0.002** | **0** | **0.002** |
| L77058 |  | **1** | **Not analyzed** | **not selected for detailed analysis** | **0** | **0** | **0** | **0** | **0** | **0** | **0** | **0.002** | **0** | **0** | **0** | **0** | **0** | **0** | **0** | **0.002** |
| L77060 |  | **2** | **Not analyzed** | **not selected for detailed analysis** | **0** | **0** | **0** | **0** | **0** | **0** | **0** | **0.002** | **0** | **0** | **0** | **0** | **0** | **0** | **0** | **0.002** |
| L77061 |  | **1** | **Not analyzed** | **not selected for detailed analysis** | **0** | **0** | **0** | **0** | **0** | **0** | **0** | **0.002** | **0** | **0** | **0** | **0** | **0** | **0** | **0** | **0.002** |
| L81283 |  | **2** | **Not analyzed** | **not selected for detailed analysis** | **0** | **0** | **0** | **0** | **0** | **0** | **0** | **0** | **0** | **0** | **0** | **0** | **0** | **0** | **0.002** | **0.002** |
| L87597 |  | **2** | **Not analyzed** | **not selected for detailed analysis** | **0** | **0** | **0** | **0** | **0** | **0.002** | **0** | **0** | **0** | **0** | **0** | **0** | **0** | **0** | **0** | **0.002** |
| L87695 |  | **3** | **Not analyzed** | **not selected for detailed analysis** | **0** | **0** | **0** | **0** | **0** | **0.002** | **0** | **0** | **0** | **0** | **0** | **0** | **0** | **0** | **0** | **0.002** |
| L87697 | **S3143** | **3** | **Not analyzed** | **identical in amino acid sequence to S3143** | **0** | **0** | **0** | **0** | **0** | **0.002** | **0** | **0** | **0** | **0** | **0** | **0** | **0** | **0** | **0** | **0.002** |
| L87698 |  | **1** | **Not analyzed** | **not selected for detailed analysis** | **0** | **0** | **0** | **0** | **0** | **0.002** | **0** | **0** | **0** | **0** | **0** | **0** | **0** | **0** | **0** | **0.002** |
| L202005 |  | **2** | **Not analyzed** | **not selected for detailed analysis** | **0** | **0** | **0** | **0** | **0** | **0.002** | **0** | **0** | **0** | **0** | **0** | **0** | **0** | **0** | **0** | **0.002** |
| L88996 |  | **2** | **Not analyzed** | **not selected for detailed analysis** | **0** | **0** | **0** | **0** | **0** | **0** | **0** | **0.001** | **0** | **0** | **0** | **0** | **0** | **0** | **0** | **0.001** |
| L228250 |  | **3** | **Not analyzed** | **not selected for detailed analysis** | **0** | **0** | **0** | **0** | **0** | **0** | **0** | **0.001** | **0** | **0** | **0** | **0** | **0** | **0** | **0** | **0.001** |
| L91153 |  | **1** | **Not analyzed** | **not selected for detailed analysis** | **0** | **0** | **0** | **0** | **0** | **0** | **0** | **0** | **0** | **0** | **0.001** | **0** | **0** | **0** | **0** | **0.001** |
| L91984 |  | **1** | **Not analyzed** | **not selected for detailed analysis** | **0** | **0** | **0** | **0** | **0** | **0** | **0** | **0** | **0** | **0** | **0** | **0** | **0** | **0** | **0.001** | **0.001** |
| L202024 |  | **1** | **Analyzed** | **Phage display–derived** | **0** | **0** | **0** | **0** | **0** | **0.001** | **0** | **0** | **0** | **0** | **0** | **0** | **0** | **0** | **0** | **0.001** |

Clones are listed in descending order of cumulative relative frequency. The Ig-15 cluster was further classified into three phylogenetic subclusters based on the branching pattern of the phylogenetic tree.

| Supplementary Table S2 Sequences and kinetic parameters in the SPR analysis of clones in the cluster | | | | | | | | | | |
| --- | --- | --- | --- | --- | --- | --- | --- | --- | --- | --- |
| Clone ID | Amino Acid Sequence | *T*m (℃) | *K*_D_ (M) | *k*_a_ (1/Ms) | SE (*k*_a_) | *k*_d_ (1/s) | SE (*k*_d_) | *X*^2^ (RU^2^) | Bit score (V+J) | Time of appearance (w) |
| Early phase generation | |  |  |  |  |  |  |  |  |  |
| L3037 | QVQLVESGGGLVQAGGSLRLSCAASGISFSINNMGWYRQAPGKQRDLVALIDKYATTNYADSVKGRFTISRDNAKNTAYLQMNSLKPEDTAVYYCNALGTWIRAGPYWGQGTQVTVSS | 67.9 ± 0.044 | 2.89 × 10^-8^** | 8195 | 21 | 2.37 × 10^-4^ | 2.3 × 10^-6^ | 2.11 | 500.6 | 3 - 4 |
| L7318 | QVQLVESGGGLVQAGGSLRLSCAASGISFSINNMGWYRQAPGKQRDLVALIDKYNTTNYADSVKGRFTISRDNAKNTAYLQMNSLKPEDTAVYYCNALGTWIRAGPYWGQGTQVTVSS | 69.7 ± 0.046 | 1.59 × 10^-8^ | 5976 | 99 | 9.51 × 10^-5^ | 2.2 × 10^-6^ | 1.73 | 494.6 | 4 |
| L11966 | QVQLVESGGGLVQAGGSLRLSCAASGISFSINNMAWYRQAPGKQRDLVALIDKYNTTNYPDSVKGRFTISRDNAKNTAYLQMNSLKPEDTAVYYCNALGTWIRAGPYWGQGTQVTVSS | 64.1 ± 0.026 | 1.43 × 10^-6^* | - | - | - | - | 0.89 | 483.6 | 4 |
| L24189 | QVQLVESGGGLVQAGGSLRLSCAASGISFSINNMGWYRQAPGKQRDLVALIDKYATTNYPDSVKGRFTISRDNAKNTAYLQMNSLKPEDTAVYYCNALGTWIRAGPYWGQGTQVTVSS | 60.6 ± 0.037 | 1.39 × 10^-6^* | - | - | - | - | 0.097 | 494.6 | 4 |
| L61790 | QVQLVESGGGLVQAGGSLRLSCAASGISFSINNMAWYRQAPGKQRDLVALIDKYNTTNYPDSVKGRFTISRDNAKNTAYLQMNSLKPEDTAVYYCNTLGTWIRAGPYWGQGTQVTVSS | 57.7 ± 0.064 | 3.76 × 10^-7^* | - | - | - | - | 0.300 | 478.6 | 4 |
| L62103 | QVQLVESGGGLVQAGGSLRLSCAASGISFSINNMGWYRQAPGKQRDLVALIDKYNTTNYTHSVKGRFTISRDNAKNTAYLQMNSLKPEDTAVYYCNALGTWIRAGPYWGQGTQVTVSS | 64.6 ± 0.034 | 2.81 × 10^-6^* | - | - | - | - | 0.221 | 483.6 | 4 |
| Middle phase generation | |  |  |  |  |  |  |  |  |  |
| L804 | QVQLVESGGGLVQAGGSLRLSCAASGISLSINNMAWYRQAPGKQRDLVALIDKYDAANSADSVKGRFTISRDNAKNTAYLQMNSLKPEDTAVYYCNAQGTWTRAGPYWGQGTQVTVSS | 59.7 ± 0.13 | 3.40 × 10^-9^ | 6.22 × 10^5^ | 3.3 × 10^3^ | 0.00211 | 1.1 × 10^-5^ | 6.14 | 461.6 | 8 - 9 |
| L1088 | QVQLVESGGGLVQAGGSLRLSCAASGISLSTNNMGWYRQAPGKQRDLVALIDKYAAANVPDSVKGRFTISRDNAKNTAYLQMNSLKPEDTAVYYCNALGTWSRAGPYWGQGTQVTVSS | 58.1 ± 0.094 | 1.39 × 10^-9^ | 5.85 × 10^5^ | 2.6 × 10^3^ | 8.16 × 10^-4^ | 2.7 × 10^-6^ | 6.08 | 455.6 | 7 - 8 |
| L1196 | QVQLVESGGGLVQAGGSLRLSCAASGIRFSSNNMGWYRQAPGKQRDLVALIDKYNTTNYVGSVKGRFSLTIDNAKNTAYLQMNSLKPEDTAVYYCNALGTWIRAGPYWGQGTQVTVSS | 64.8 ± 0.015 | 2.38 × 10^-7^ | - | - | - | - |  | 428.6 | 8-9 |
| L2362 | QVQLVESGGGLVQAGGSLRLSCAASGISFSINNMGWYRQAPGKQRDLVALIDKYNTTNIPDSVKGRFTISRDDAKNTAYLQMNSLKPEDTAVYYCNALGTWTRAGPYWGQGTQVTVSS | 60.5 ± 0.16 | 2.58 × 10^-8^ | 8.96 × 10^5^ | 7.7 × 10^3^ | 0.023 | 2.2 × 10^-4^ | 3.95 | 472.6 | 8 |
| L3585 | QVQLVESGGGLVQAGGSLRLSCAASGINLSINNVAWYRQAPGKQRDLVALIDKYDTANHADSVKGRFTISRDNAKNTAYLQINSLKPEDTAVYYCNALGTWIRAGPYWGQGTQVTVSS | 67.9 ± 0.087 | 4.42 × 10^-9^ | 4.68 × 10^5^ | 2.1 × 10^3^ | 0.0021 | 8.8 × 10^-6^ | 4.58 | 466.6 | 8 |
| L4105 | QVQLVESGGGLVQAGGSLRLSCAASGISLRINNLAWYRQAPGKQRDLVALIDKYDTANLPDSMKGRFTISRDNAKNTAYLEMNSLKPEDTAVYYCNALGTWIEPGPYWGQGTQVTVSS | 63.6 ± 0.12 | 7.09 × 10^-9^ | 4.87 × 10^5^ | 2.2 × 10^3^ | 0.0034 | 1.6 × 10^-5^ | 3.07 | 439.6 | 8 - 10 |
| L4899 | QVQLVESGGGLVQAGGSLRLSCAASGISFSINNMGWYRQAPGKQRDLVALIDKYNTTNYPDSVKGRFSLSIDNAKNTAYLQMNSLKPEDTAVYYCNALGTWIRAGPYWGQGTQVTVSS | 62.6 ± 0.43 | 6.65 × 10^-7^* | - | - | - | - | 5.08 | 472.6 | 8 - 9 |
| L6805 | QVQLVESGGGLVQAGGSLRLSCAASGISFSGNNMGWYRQAPGKQRDLVALIDKYDTANIPDSVKGRFTISRDNAKNTAYLQMNSLKPEDTAVYYCNALGTWIRPGPYWGQGTQVTVSS | 60.4 ± 0.15 | 3.92 × 10^-9^ | 7.71 × 10^5^ | 3.7 × 10^3^ | 0.0030 | 1.5 × 10^-5^ | 10.6 | 466.6 | 7 - 10 |
| L15101 | QVQLVESGGGLVQAGGSLRLSCAASGISFSINNMGWYRQAPGKQRDLVALIDKYNTTNYPDSLKGRFTISRDNAKNTAYLQMNSLKPEDTAVYYCNALGTWIRAGPYWGQGTQVTVSS | 60.6 ± 0.061 | 1.58 × 10^-6^* | - | - | - | - | 1.47 | 483.6 | 5 - 6 |
| L15139 | QVQLVESGGGLVQAGGSLRLSCAASGISFSGNNMGWYRQAPGKQRDLVALIDKYDTTNYPDSVKGRFTISRDNAKNTAYLQMNSLKPEDTAVYYCNALGTWIRAGPYWGQGTQVTVSS | 50.0 ± 0.15 | 2.84 × 10^-7^* | - | - | - | - | 4.16 | 483.6 | 6 |
| L15144 | QVQLVESGGGLVQAGGSLRLSCTASGITLSINNMAWYRQAPGKQRDLVALIDKYNTTNYADSVKGRFSISIDNAENTAYLQMNSLKPEDTAVYYCNALGTWIRAGPYWGQGTQVTVSS | 66.0 ± 0.031 | 1.89 × 10^-7^ | 7398 | 2.1 × 10^2^ | 0.0014 | 2.9 × 10^-5^ | 3.2 | 450.6 | 6 |
| L19817 | QVQLVESGGGLVQAGGSLRLSCAASGISFSINNMAWYRQAPGKQRDLVALIDKYNTTNYADSVKGRFSLSRDNAKNTAYLQMNSLKPEDTAVYYCNALGTWIRAGPYWGQGTQVTVSS | 67.4 ± 0.031 | 2.82 × 10^-6^* | - | - | - | - | 0.095 | 476.8 | 6 - 8 |
| L21793 | QVQLVESGGGLVQAGGSLRLSCAASGISFSINNVAWYRQAPGKQRDLVALIDKYDTANHADSVKGRFTISRDNAKNTAYLQMNSLKPEDTAVYYCNALGTWIRAGPYWGQGTQVTVSS | 64.2 ± 0.050 | 3.41 × 10^-8^ | 4.75 × 10^5^ | 2.6 × 10^3^ | 0.016 | 9.6 × 10^-5^ | 3.96 | 478.6 | 5-7 |
| L202024 | QVQLVESGGGLVQAGGSLRLSCAASGISFAINNVAWYRQAPGKQRDLVALIDKYDTGNIADSVKGRFSISRDNAKNTAYLQMNSLKPEDTAVYYCNALGTWIRAGPYWGQGTQVTVSS | 66.7 ± 0.020 | 3.82 × 10^-9^ | 4.40 × 10^5^ | 1.2 × 10^3^ | 0.0016 | 4.1 × 10-^6^ | 6.31 | 433.6 | 6 |
| S1146 | QVQLVESGGGLVQAGGSLRLSCAASGISFSINNMGWYRQAPGKQRDLVALIDKYNTTNYVDSVKGRFSLSIDNAKNTAYLQMNSLKPEDTAVYYCNALGTWIRAGPYWGQGTQVTVSS | 62.5 ± 0.035 | 6.47 × 10^-7^* | - | - | - | - | 0.205 | 466.6 | 8 - 9, 12 - 13 |
| S1156 | QVQLVESGGGLVQAGGSLRLSCAASGISFSINNMGWYRQAPGKQRDLVALIDKYNTTNYVDSVKGRFTISIDNAKNTAYLQMNSLKPEDTAVYYCNALGTWIRAGPYWGQGTQVTVSS | 65.6 ± 0.053 | 6.73 × 10^-7^* | - | - | - | - | 0.052 | 478.6 | 8 |
| S1908 | QVQLVESGGGLVQAGGSLRLSCAASGITLSINNVAWYRQAPGKQRDLVALIDKYASANVPDSVKGRFTISRDNAKNTAFLQMNSLKPEDTAVYYCNALGTWTRAGPYWGQGTQVTVSS | 61.6 ± 0.066 | 1.17 × 10^-9^ | 7.44 × 10^5^ | 3.6 × 10^3^ | 8.71 × 10^-4^ | 3.2 × 10^-6^ | 3.60 | 439.6 | 8 |
| S5942 | QVQLVESGGGLVQAGGSLRLSCAASGISFRDNNMAWYRQAPGKQRDLVALIDKYATANVPDSVKGRFTISRDNAKNTAYLQMNSLKPEDTAVYYCNALGTWTRAGPYWGQGTQVTVSS | 61.3 ± 0.075 | 2.35 × 10^-9^ | 7.19 × 10^5^ | 3.3 × 10^3^ | 0.0017 | 7.0 × 10^-6^ | 3.60 | 433.6 | 8 |
| Late phase generation | |  |  |  |  |  |  |  |  |  |
| L926 | QVQLVESGGGLVQAGGSLRLSCAASGISLRDDNMGWYRQAPGKQRDLVALIDKYNTTNYVDSVKGRFSLSIDNAKNTAYLQMNSLKPEDTAVYYCNALGTWIRAGPYWGQGTQVTVSS | 61.8 ± 0.025 | 1.83 × 10^-8^ | 7.4 × 10^4^ | 5.0 × 10^2^ | 0.0013 | 7.3 × 10^-6^ | 1.08 | 428.1 | 12 - 15 |
| L1332 | QVQLVESGGGLVTAGGSLRLSCAASGISLRINNVAWYRQAPGKQRDLVALIDKYDSANSPDSMEGRFTISRDNAKNTAYLEMNSLKPEDTAVYYCNALGTWIEPGPYWGQGTQVTVSS | 65.6 ± 0.057 | 8.08 × 10^-10^** | 6.3 × 10^5^ | 9.4 × 10^2^ | 5.11× 10^-4^ | 1.6 × 10^-6^ | 1.55 | 411.6 | 14 - 15 |
| L7813 | QVQLVESGGGLVQAGGSLRLSCAASGISFSRNNMAWYRQAPGKQRDLVALIDKYDTANVADIVKGRFTISRNNAGNTAYLQMNSLKPEDTAVYYCNALGTWIRAGPYWGQGTQVTVSS | 61.1 ± 0.055 | 1.39 × 10^-9^ | 1.78 × 10^6^ | 4.9 × 10^3^ | 0.0015 | 7.3 × 10^-6^ | 7.26 | 433.6 | 13 |
| L14600 | QVQLVESGGGLVQAGGSLRLSCAASGITLSANNMAWYRQAPGKQRVLVALIDKYDTANVPDFVKGRFTISRDNAKNTAFLQMNSLEPEDTAVYYCNALGTWTRAGPYWGQGTQVTVSS | 46.5 ± 0.13 | 5.51 × 10^-10^** | 6.92 × 10^5^ | 4.0 × 10^3^ | 4.08 × 10^-4^ | 1.9 × 10^-6^ | 3.13 | 401.1 | 15 |
| L36104 | QVQLVESGGGLVQAGGSLRLSCAASGISLRNNNMGWYRQAPGKQRDLVALIDKYNTTNYVDSVKGRFSLSIDNAKNTAYLQMNSLKPEDTAVYYCNALGTWIRAGPYWGQGTQVTVSS | 49.9 ± 0.092 | 2.45 × 10^-7^* | - | - | - | - | 0.394 | 445.1 | 13 |
| S543 | QVQLVESGGGLVQAGGSLRLSCAASGISFSDNNVAWYRQAPGKQRELVALIDKYNAANSPDFLKGRFTISRDNAKNTAYLQMNSLTPEDTAVYYCNVLGTWTRAGPYWGQGTHVTVSS | 61.7 ± 0.14 | 4.11 × 10^-10^** | 7.00 × 10^+5^ | 1.9 × 10^3^ | 2.87 × 10^-4^ | 2.6 × 10^-6^ | 2.09 | 425.1 | 13, 15 |
| S3146 | QVQLVESGGGLVQAGGSLRLSCVASGISFSDNNMAWYRQAPGKQRELVALIDKYNAANSPDFLKGRFTISRDNAKNTAYLQMNSLKPEDTAVYYCNTLGTWTRAGPYWGQGTQVTVSS | 61.4 ± 0.092 | 2.67 × 10^-10^** | 7.58 × 10^5^ | 1.9 × 10^3^ | 2.87 × 10^-4^ | 2.6 × 10^-6^ | 2.09 | 428.6 | 13 - 14 |
| S3449 | QVQLVESGGGLVQAGGSLRLSCAASGISISDNNMAWYRQAPGKQRELVALIDKYNAANSPDSMKGRFIISRDTAKNTAYLQMNSLKPEDTAVYYCNALGTWIRAGPYWGQGTQVTVSS | 53.4 ± 0.11 | 2.41 × 10^-10^** | 6.97 × 10^5^ | 1.2 × 10^3^ | 1.68 × 10^-4^ | 1.8 × 10^-6^ | 2.81 | 417.6 | 13, 15 |
| S4008 | QVQLVESGGGLVQAGGSLRLSCAASGISFSDNNVAWYRQAPGKQRELVALIDKYNAANSPDFVKGRFTISRDNAKNTAYLQMNNLTPEDTAVYYCNTLGTWTRAGPYWGQGTHVTVSS | 57.7 ± 0.18 | 9.32 × 10^-10^** | 5.19 × 10^5^ | 7.4 × 10^2^ | 4.84 × 10^-4^ | 1.5 × 10^-6^ | 1.34 | 423.1 | 13 |
| S4511 | QVQLVESGGGLVQAGGSLRLSCAASGISFSGVNVAWYRQAPGKQRELVALIDKYNAANSPDFLKGRFTISRDNAENTAYLQMNSLTPEDTAVYYCNALGTWTRAGPYWGQGTHVTVSS | 54.9 ± 0.098 | 4.15 × 10^-10^** | 4.94 × 10^5^ | 9.4 × 10^2^ | 2.05 × 10^-4^ | 2.0 × 10^-6^ | 2.37 | 412.1 | 13 |
| S4727 | QVQLVESGGGLVQAGGSLRLSCAASGINFSGNNMAWYRQAPGKQRELVALIDKYNAANSPDFLKGRFTISRDNAKNTAYLQMNSLKPEDTAVYYCNALGTWSRAGPYWGQGTQVTVSS | 65.1 ± 0.052 | 3.19 × 10^-10^** | 7.22 × 10^5^ | 8.7 × 10^2^ | 2.3 × 10^-4^ | 1.3 × 10^-6^ | 1.28 | 433.6 | 13 |

*Steady State Affinity analysis

**Capture method

Supplementary Table S3 Data collection and refinement statistics

| Trastuzumab F_ab_: L202024 | | |
| --- | --- | --- |
| Data collection | | |
| Space group | C222_1_ |  |
| a, b, c (Å) | 129.03, 168.40, 60.76 |  |
| Resolution (Å) | 3.11-3.00 |  |
| R-merge | 0.233 [1.10] |  |
| I/sigmaI | 12.3 [2.3] |  |
| Redundancy | 7.2 [7.2] |  |
| Refinement | | |
| Resolution (Å) | 3.0 |  |
| No. of reflections [work/test] | 13760/1357 |  |
| Rwork/Rfree | 21.0/27.9 |  |

Supplementary Table S4 Hydrogen bonds between Trastuzumab-F_ab_ and L202024

| Source atoms | | Target atoms | | Distance (Å) |
| --- | --- | --- | --- | --- |
| Trastuzumab-F_ab_  Heavy chain | | L202024 | |  |
| 13Q | OE1 | 49Q | NE2 | 3.3 |
| 13Q | NE2 | 49Q | NE2 | 3.2 |
| 122S | OG | 52L | O | 2.7 |
| 122S | O | 110W | NE1 | 2.9 |
| 123T | O | 69D | N | 3.0 |
| 125G | N | 69D | N | 3.0 |
| 150K | NZ | 64T | OG1 | 3.2 |
| 179S | O | 38N | ND2 | 2.8 |
| 180S | O | 110W | N | 3.0 |
| Trastuzumab-F_ab_  Light chain | | L202024 | |  |
| 128G | O | 63D | O | 3.4 |
| 129T | OG1 | 59Y | O | 2.5 |
|  |  | 59Y | OG1 | 2.7 |

Underlined amino acids were identified as key interacting residues in the crystal structure of the F_ab_–L202024 complex.

Supplementary Table S5 Reduction in ASA during complex formation per amino acid residue

| CL | | CH1 | | L202024 | |
| --- | --- | --- | --- | --- | --- |
| Residue | Area (Å^2^) | Residue | Area (Å^2^) | Residue | Area (Å^2^) |
| 124Q | 0.5 | 13Q | 33.2 | 38N (CDR-1) | 21.6 |
| 127S | 30.0 | 119S | 12.3 | 49Q (FR-2) | 38.0 |
| 128G | 17.6 | 120S | 4.8 | 51D (FR-2) | 2.8 |
| 129T | 33.6 | 121A | 25.3 | 52L (FR-2) | 19.3 |
| 157G | 18.9 | 122S | 80.2 | 55L (FR-2) | 8.4 |
| 158N | 17.5 | 123T | 33.2 | 57D (CDR-2) | 30.0 |
| 180T | 42.2 | 124K | 69.0 | 58K (CDR-2) | 29.6 |
| 181L | 4.1 | 125G | 8.1 | 59Y (CDR-2) | 141.4 |
| 182S | 38.7 | 129F | 0.4 | 63D (CDR-2) | 58.0 |
| 183K | 0.4 | 150K | 17.2 | 64T (CDR-2) | 64.0 |
| 184A | 7.1 | 151D | 25.5 | 65G (CDR-2) | 4.9 |
|  |  | 153F | 18.8 | 66N (FR-3) | 54.1 |
|  |  | 177L | 16.4 | 67I (FR-3) | 22.3 |
|  |  | 178Q | 2.7 | 68A (FR-3) | 9.9 |
|  |  | 179S | 92.5 | 69D (FR-3) | 77.3 |
|  |  | 180S | 73.6 | 70S (FR-3) | 4.3 |
|  |  | 181G | 35.5 | 72K (FR-3) | 6.1 |
|  |  | 182L | 33.8 | 109T (CDR-3) | 22.4 |
|  |  | 210S | 28.2 | 110W (CDR-3) | 121.9 |
|  |  | 212T | 0.2 | 112I (CDR-3) | 86.0 |
| Total | 210.6 | Total | 610.9 | Total | 822.3 |

Underlined amino acids indicate residues that showed large changes in the ASA of L202024 upon binding to F_ab_.

Supplementary Table S6 Reduction in ASA during complex formation per molecule.

|  | Individual (Å^2^) | Complex (Å^2^) | Change (%) |
| --- | --- | --- | --- |
| CL | 9668.8 | 9458.2 | 2.18 |
| CH1 | 9612.4 | 9001.5 | 6.36 |
| L202024 | 6289.6 | 5467.3 | 13.07 |

Supplementary Table S7 Evaluation of binding kinetics using antigen immobilization and capture methods.

| ID | F_ab_ immobilization method | | | F_ab_ capture method | | |
| --- | --- | --- | --- | --- | --- | --- |
|  | *K*_D_(M) | *k*_a_(1/Ms) | *k*_d_(1/s) | *K*_D_(M) | *k*_a_(1/Ms) | *k*_d_(1/s) |
| L11966 | 1.43 ×10^-6^* | - | - | 1.96 × 10^-6^* | - | - |
| L7318 | 1.59 × 10^-8^ | 5976 | 9.51 × 10^-5^ | 2.43 × 10^-8^ | 9645 | 2.43 × 10^-4^ |
| L804 | 3.40 × 10^-9^ | 6.22 × 10^5^ | 0.00211 | 2.57 ×10-^9^ | 8.53×10^5^ | 0.00219 |
| L1088 | 1.39 ×10^-9^ | 5.85 × 10^5^ | 8.16 ×10-^4^ | 1.53 ×10-^9^ | 7.86×10^5^ | 0.00121 |
| L4105 | 7.09×10^-9^ | 4.87×10^5^ | 0.0034 | 4.07 ×10^-9^ | 7.79×10^5^ | 0.0036 |
| S5942 | 2.35×10^-9^ | 7.19×10^5^ | 0.0017 | 4.21 ×10^-9^ | 6.30×10^5^ | 0.0026 |

*Steady State Affinity analysis
